# Potential of Quantitative α‑Amylase or Trypsin Inhibition by Refined and Whole Wheat and Einkorn Using High-Performance Thin-Layer Chromatography–NanoGIT versus Conventional Spectrophotometry

**DOI:** 10.1021/acs.jafc.5c14009

**Published:** 2026-02-20

**Authors:** Isabel Müller, Ilka Scheibelhut, Gertrud E. Morlock

**Affiliations:** Chair of Food Science, Institute of Nutritional Science, and Interdisciplinary Research Centre for Biosystems, Land Use and Nutrition, 9175 Justus Liebig University Giessen, Heinrich-Buff-Ring 26-32, 35392 Giessen, Germany

**Keywords:** α-amylase/trypsin inhibitor (ATI), on-surface metabolization, enzyme inhibition assay, nonceliac wheat sensitivity

## Abstract

The current analysis of α-amylase/trypsin inhibitors (ATIs) is complicated due to the missing link between inhibition potential and inhibitors. Two on-surface assays (nanoGIT) were developed to quantify the inhibition of α-amylase and trypsin by flour extracts of refined wheat, whole wheat, and einkorn, followed by direct analysis (on the same surface) of the metabolisation products via high-performance thin-layer chromatography (HPTLC). The HPTLC–nanoGIT (amylolysis inhibition)–FLD/Vis revealed a lower α-amylase inhibition of einkorn than refined or whole wheat, whereby both latter wheat types showed no differences. The HPTLC–nanoGIT (proteolysis inhibition)–Vis confirmed only partially the 100% inhibition at high extract concentrations of the three cereal types obtained by the spectrophotometric trypsin inhibition assay. The HPTLC–nanoGIT workflows not only confirmed the inhibitory effects obtained by spectrophotometric assays but also provided substantial resolution of saccharide-specific interactions and specific profile patterns of the released individual metabolisation products.

## Graphical abstract

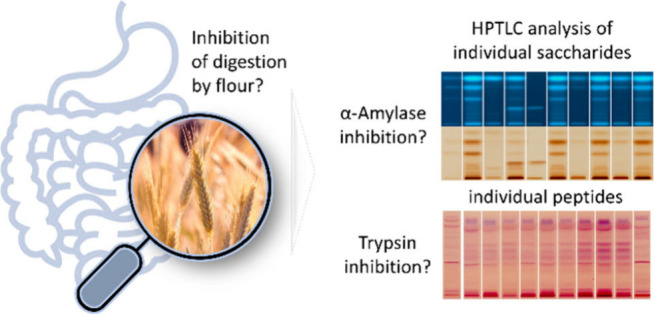

## 1 Introduction

α-Amylase/trypsin inhibitors (ATIs) are naturally occurring proteins found in plant seeds and inhibit both digestive enzymes, which are essential for breaking down carbohydrates and proteins in the human body.^1^ They account for 2–4% of the total proteins in wheat, and the 19 ATIs known to date occur in monomeric, dimeric, or tetrameric form.^2,3^ ATIs have been shown to have a negative impact on digestive health and can contribute to several health issues, such as gastrointestinal distress^1,4^ and inflammation.^5,6^ In numerous studies, ATIs were discussed as potential triggers of nonceliac wheat sensitivity (NCWS), a disease with a widely spread but also highly varying range of prevalence (0.5–15%).^7–10^ The water-soluble low molecular weight proteins could be a concern for consumers, as they are suspected to be heat resistant and could resist food processing such as baking and cooking.^8,10,11^ Contrary studies, mainly dealing only with α-amylase activity, stated a reduction in activity and thus less inhibition after heat treatment.^12,13^ The exact cause of NCWS is not well understood, and other triggers such as gluten-related gliadins and/or fermentable oligosaccharides, disaccharides, monosaccharides, and polyols (FODMAPs) are discussed as well.^10^ Sparse research has led to an upcoming interest in ATIs as potential disruptors of the human digestive system.

Several analytical approaches have been studied to identify ATIs in cereals and elucidate their specific roles in gastrointestinal complaints. One is tandem mass spectrometric analysis to identify and/or quantify the content of already known ATIs in cereal products.^14–23^ Another is the application of spectrophotometric methods to analyze the inhibition as a sum value.^21,22,24^ These techniques have in common that they identify the inhibitory potential or quantify already known inhibitors, but do not directly link the observed inhibition with specific inhibitors. Very rarely, both techniques were combined,^25^ presumably due to their huge effort and costs.

In our previous study,^26^ the potential of high-performance thin-layer chromatography (HPTLC) for the analysis of matrix-rich cereal extracts containing ATIs was demonstrated for the first time. It revealed a massively disturbing matrix load after standardized extraction. The current study aimed to streamline and improve the previously developed HPTLC screening.^26^ It was hypothesized that the workflow can be performed completely on the surface in one step together (HPTLC–nanoGIT), and that inhibition by ATIs can be analyzed quantitatively. It shall result in a better alternative method for the assessment of inhibitors besides vulnerable spectrophotometric methods.

## 2 Materials and Methods

### 2.1 Chemicals and Materials

Acarbose (≥95%), d-(+)-glucose (≥99.5%), maltotriose hydrate (97%), 4-aminobenzoic acid (≥99%), d-(+)-maltose monohydrate (≥99%), trypsin inhibitor from *Glycine max* (90%), casein from bovine milk (technical grade), pyridine (≥99%), ammonium hydroxide solution (25%, puriss p.a.), *N*_α_-benzoyl-l-arginin-4-nitroanilid-hydrochlorid (L-BAPA, ≥98%), sodium acetate trihydrate (≥99.0%), magnesium chloride hexahydrate (≥99.0%), 2-naphthol (>99%), α-amylase from hog pancreas (45.5 U/mg) and trypsin from bovine pancreas (97%; 10,000 *N*_α_-benzoyl-l-arginine ethyl ester, i.e., BAEE U/mg protein) were purchased from Sigma-Aldrich Fluka (Steinheim, Germany). *o-*Phosphoric acid (85%, p.a.) was from Th. Geyer (Renningen, Germany). 2-Propanol (≥99.8%), calcium chloride dihydrate (≥98%), sodium dihydrogen phosphate monohydrate (≥98%), disodium hydrogen phosphate (≥99%), hydrochloric acid (37%, p.a.), sodium hydroxide (≥99%), alkali-soluble casein (≥95%), dimethyl sulfoxide (≥99.8%), molecular sieve (0.3 nm, type 564, beads), sulfuric acid (≥95%), and tris­(hydroxymethyl)­aminomethane (Tris, ≥ 99.9%) were from Carl Roth (Karlsruhe, Germany). Acetic acid (99–100%), acetonitrile (≥99.9%), potassium iodide (puriss p.a.), and starch soluble (analytical grade) were from Riedel-de Haen (Seelze, Germany). Acetone (≥99.9%), sodium chloride (≥99%), and 0.45 μm cellulose acetate membrane syringe filters were purchased from VWR International (Darmstadt, Germany). *n*-Hexane (≥96%), ninhydrin (analytical grade), HPTLC plates silica gel 60 (20 cm × 10 cm), Novagen D-Tube Dialyzer Mega (10 mL) with molecular weight cutoff (MWCO) at 3.5 kDa, and iodine (double sublimated, p.a.) were obtained from Merck (Darmstadt, Germany). 2-Butanol (99%) was delivered by Alfa Aesar (Kandel, Germany). Ethanol (≥99.8%) was supplied by Thermo Fisher Scientific (Geel, Belgium). Ammonium bicarbonate (99%) was obtained from Acros Organics (Morris Plains, NJ, USA). Bidistilled water was produced by a Heraeus Destamat B-18E (Thermo Fisher Scientific, Dreieich, Germany). Three flour samples were purchased from local supermarkets. Flours of conventional unbleached, refined wheat type 405 (Belbake, Lidl) and organic whole grain wheat (Rewe Bio, Rewe) were both produced by Friesinger Mühle (Bad Wimpfen, Germany), whereas einkorn was produced by Spielberger (Brackenheim, Germany).

### 2.2 Preparation of Solutions

#### 2.2.1 HPTLC–NanoGIT (Amylolysis Inhibition)–FLD/Vis

As a positive control (PC), acarbose (1 mg/mL) was dissolved in water. Soluble starch (10 mg/mL) was dissolved in water and heated in a boiling water bath until a clear solution was obtained. The solution was used the next day or stored at 4 °C. It was warmed up to about 20 °C for analysis; the clarity of the solution was proven, and if needed, reheated to 100 °C (MR Hei-Standard, Heidolph Instruments, Schwabach, Germany) before application. α-Amylase (5 mg/mL) was dissolved in 20 mM sodium acetate trihydrate and 7 mM sodium chloride to obtain a solution of 227.5 U/mL. Sodium chloride-containing phosphate buffer (1.3 mM phosphate buffer pH 7 with 150 mM sodium chloride) was prepared by diluting (1:8 *V*/*V*) a 10 mM phosphate buffer (0.5 mg/mL disodium hydrogen phosphate and 0.9 mg/mL sodium dihydrogen phosphate monohydrate) and adding sodium chloride (0.9 mg/mL). For the *p-*aminobenzoic acid reagent, 2 g of 4-aminobenzoic acid was dissolved in 252 mL acetic acid/water/acetone/*o*-phosphoric acid 1:1:3:0.04 (*V*/*V*/*V*/*V*). For the 2-naphthol reagent, 2 g of 2-naphthol was dissolved in ethanol/50% sulfuric acid 24:1 (*V*/*V*).

#### 2.2.2 Spectrophotometric α-Amylase Inhibition Assay for Comparison

α-Amylase (0.5 mg/mL, 22.8 U/mL) was dissolved in a 1:1 (*V*/*V*) mixture of water and sodium phosphate buffer (20 mM, pH 6.9, mixture of 1.1 mg/mL disodium hydrogen phosphate, 1.7 mg/mL sodium dihydrogen phosphate monohydrate, and 0.4 mg/mL sodium chloride). Iodine/iodide solution (0.3 mg/mL iodine and 0.15 mg/mL potassium iodide in water) was prepared, and as mentioned, a soluble starch solution (1 mg/mL).

#### 2.2.3 In-Vial Trypsin Inhibition Assay for Method Development

Trypsin (0.33 mg/mL, i.e., 33,000 BAEE U/mL) was dissolved in 0.6 M calcium chloride, pH 3, and adjusted to pH 8 by mixing 100 μL with 1 μL 1 M sodium hydroxide. Trypsin inhibitor (0.05 and 0.5 mg/mL) was dissolved in water. Casein (20 mg/mL) was dissolved in 25 mM ammonium bicarbonate by mixing either alkali-soluble casein or casein from bovine milk dropwise with 1 M sodium hydroxide, followed by ultrasonification (no heat, Sonorex Digiplus, Bandelin, Berlin, Germany) until pH 11 was reached. Alkali-soluble casein dissolved completely until clarity and needed less sodium hydroxide than casein from bovine milk, which remained turbid. The required pH of 7 was adjusted dropwise using 1 M hydrochloric acid. Below pH 6, casein precipitated and was resolved by adjusting the pH to basic.

#### 2.2.4 HPTLC–NanoGIT (Proteolysis Inhibition)–Vis

Trypsin (0.2 mg/mL, i.e., 2,000 BAEE U/mL) was dissolved in water, adjusted to pH 3 using 1 M hydrochloric acid, and adjusted before analysis to pH 8 using 1 M sodium hydroxide. Trypsin inhibitor (0.5 mg/mL) was dissolved in water. Casein (20 mg/mL) was dissolved as mentioned. A saturated solution of magnesium chloride (33% relative humidity) was prepared in water (544 g/L). For the ninhydrin reagent, ninhydrin (2 mg/mL) was dissolved in ethanol/acetic acid 12:1 (*V*/*V*).

#### 2.2.5 Spectrophotometric Trypsin Inhibition Assay for Comparison

As a stock solution (0.5 mg/mL), L-BAPA (7.5 mg) was dissolved in dimethyl sulfoxide (0.1 mL) and filled up to 15 mL with tris-calcium chloride buffer (50 mM tris +5 mM calcium chloride monohydrate, pH 8.2 ± 0.1). After being dissolved in dimethyl sulfoxide, the solution became turbid; however, with the addition of tris-calcium chloride buffer, it turned pale yellow. The solution was diluted with tris-calcium chloride buffer to obtain the L-BAPA working solution (0.27 mg/mL). The solution was stable at 4 °C for one day. Trypsin (0.0135 mg/mL) was dissolved in a 5 M calcium chloride solution.

### 2.3 ATI Extraction and Removal of Saccharides

ATI extraction was performed modified^26^ according to Call et al.^22^ Briefly, flour (1 g) was weighed into a 15 mL reaction tube and defatted twice with *n*-hexane (10 mL each) by mixing and in-between centrifugation (3000*g*, 1 min). The supernatant was discarded, and the remaining *n*-hexane was removed by a flow of nitrogen (TH 26, HLC BioTech, Bovenden, Germany). Sodium chloride-containing phosphate buffer (pH 7, 5 mL) was added to each flour. The suspension was vortexed for 10 min using a multitube holder (Vortex Genie 2, Scientific Industries, New York City, NY, USA) and centrifuged at 3000 *× g* for 10 min. Extraction with buffer was repeated, and both supernatants were combined. All extracts (0.1 g flour/mL buffer) were filtered with a 0.45-μm cellulose acetate membrane syringe filter, resulting in clear extracts.

For removal of intrinsic saccharides, flour extracts or α-amylase solution (5 mg/mL, i.e., 227.5 U/mL) were dialyzed (each 5 mL) against stirred water (2 L) using a D-Tube Dialyzer Mega (MWCO 3.5 kDa), prepared as specified by the manufacturer. The conductivity of the permeate was measured continuously (LF92, WTW, Weilheim, Germany) and renewed every 2 h. An aliquot (0.2 mL) of the retentate (flour extract or α-amylase solution) was withdrawn after each cycle as a process control to check the progress until dialysis was successful. All purified solutions were stored at −20 °C. After every second renewal (every 4 h), the initial salt load of the retentate (20 mM sodium acetate and 7 mM sodium chloride) was restored by adding the corresponding salt amount, taking into account the retentate withdrawal (0.4 mL) as follows:

added salt amount(g)=salt molarity(mol/L)×residual
retentate volume(L)×molecular weight of salt(g/mol)

### 2.4 Quantification via HPTLC–NanoGIT (Amylolysis Inhibition)–FLD/Vis

Acarbose solution (5 μL/band, 1 mg/mL) was used as a PC to inhibit amylolysis, and each purified flour extract (2–11 μL/band) was applied twice onto the HPTLC plate silica gel 60 (20 cm × 10 cm). One was oversprayed onto the purified α-amylase solution (5 μL/band, 227.5 U/mL), and the other was used as a reference blank. Soluble starch solution (2 μL/band, 10 mg/mL) was applied on bands intended for amylolysis. The negative control (NC) did not contain acarbose or the extract for maximal amylolysis. All substances were applied once with the following settings: band length 7 mm, track distance 10 mm, distance from the lower edge 10 mm and left edge 15 mm, dosage speed 50 nL/s, filling speed 8 μL/s, filling vacuum time 4 s, and a syringe volume of 25 μL (Automatic TLC Sampler ATS4, CAMAG, Muttenz, Switzerland). The plate, except the application zone, was covered with a cut HPTLC plate^27^ (layer upward, 20 cm × 8.5 cm) and wetted with a 0.1 M sodium chloride solution by piezoelectrical spraying (2.5 mL, yellow nozzle, level 6, Derivatizer, CAMAG). The covered plate was incubated at 37 °C in a humid plastic box filled with 70 mL water (26.5 cm × 16 cm × 10 cm, ABM, Wolframs-Eschenbach, Germany) for 30 min and then dried at 120 °C for 10 min (TLC Plate Heater III, CAMAG). The development was performed in a twin-trough chamber (20 cm × 10 cm) with 8 mL acetonitrile/water/2-propanol 3:1:1 (*V*/*V*/*V*) up to 70 mm (taking 15 min). For a derivatization reagent sequence, the plate was piezoelectrically sprayed (4 mL, yellow nozzle, level 6, Derivatizer) with the *p*-aminobenzoic acid reagent, followed by heating (140 °C, 5 min, TLC Plate Heater III). The chromatogram was detected at FLD 366 nm (TLC Visualizer 2, CAMAG) and for densitometric fluorescence measurement at 366/>400 nm (slit 4.0 mm × 0.2 mm, mercury lamp, TLC Scanner 4, CAMAG). The same plate was derivatized with the 2-naphthol reagent by piezoelectric spraying (4 mL, yellow nozzle, level 6, Derivatizer), followed by heating (120 °C, 5 min). The chromatogram was detected at white light illumination (Vis, remission–transmission mode; TLC Visualizer 2) and measured densitometrically at 500 nm (absorbance measurement, slit 4.0 mm × 0.2 mm, deuterium/tungsten lamp, TLC Scanner 4). Instrument operation and data evaluation were performed using visionCATS software (version 3.1, CAMAG).

### 2.5 Comparison with a Spectrophotometric α-Amylase Inhibition Assay

For the iodine/iodide α-amylase inhibition assay,^28^ 400 μL of α-amylase (22.8 U/mL, 0.5 mg/mL) and 300 μL of PC acarbose (1 mg/mL) or nonpurified flour extract from refined wheat were mixed in a 2 mL reaction tube and preincubated at 37 °C for 10 min. Soluble starch (400 μL, 1 mg/mL) was added, and the mixture was incubated at 50 °C for 30 min. For the NC (no flour extract), non-amylolysed acarbose, and flour extract serving as blank, sodium phosphate buffer (pH 6.9) was added to reach the final volume. The enzymatic reaction was stopped with 200 μL of 1 M hydrochloric acid solution, and 900 μL iodine/iodide solution was added. Absorbance of the iodine–starch complex was measured at 630 nm using a spectrophotometer (M501, Camspec, Londorf, Germany). For alternative measurement, a miniaturized combined hand-held cell counter and spectrometer (Fluidlab R-300, Anvajo, Dresden, Germany) was used.

### 2.6 In-Vial Trypsin Inhibition Assay for Method Development

The in-vial trypsin inhibition assay^26^ was modified. The flour extract (100 and 150 μL) or trypsin inhibitor (168 μL of 0.05 mg/mL and 25 μL of 0.5 mg/mL) was preincubated (10 min) with trypsin (12.1 μL, 0.33 mg/mL, pH 8) in a sample vial with 200 μL micro insert followed by the incubation with casein (20 μL, 20 mg/mL) for 30 min. As NC, trypsin and casein were incubated without trypsin inhibitor or extract. Trypsin inhibitor, trypsin, and flour extracts in the mentioned volumes, but without proteolysis, were used as reference blanks. If necessary, the final assay volume was adjusted to 200 μL by adding 25 mM ammonium bicarbonate. The final concentrations of trypsin and casein were 0.02 and 2 mg/mL, respectively. The enzyme-to-substrate ratio (E/S) was 1:100 (4 μg trypsin and 400 μg casein solutions). The enzyme-to-trypsin inhibitor ratio (E/I) was 1:2 and 1:3 (4 μg trypsin solution and 8.4 μg or 12.5 μg trypsin inhibitor solution). Trypsin inhibition was evaluated via HPTLC-Vis by application of the proteolyzed and non-proteolyzed samples and blank references (7 μL/band each) onto an HPTLC silica gel 60 plate (20 cm × 10 cm) with the same application settings (except for a band length of 8 mm and distance from the left edge of 18 mm) as mentioned for the HPTLC–nanoGIT (amylolysis inhibition)–FLD/Vis. The plate was automatically developed with 10 mL of 2-butanol/pyridine/ammonia (25%)/water (10:17:5:13, *V*/*V*/*V*/*V*) up to 50 mm (Automatic Development Chamber ADC2, CAMAG) and derivatized with the ninhydrin reagent (2 mL, yellow nozzle, Derivatizer) and heated (120 °C, 5 min). The resulting chromatograms were detected at Vis (remission–transmission mode).

### 2.7 Screening via HPTLC–NanoGIT (Proteolysis Inhibition)–Vis

Trypsin (4 μL/band, 0.2 mg/mL) was applied on the HPTLC plate silica gel 60 (20 cm × 10 cm), where proteolysis was intended. Trypsin inhibitor solution (5 μL/band, 1 mg/mL) used as PC, or flour extract (non-purified, 2, 4, and 7 μL/band) was applied twice, whereby one was applied on top of the trypsin and the other was used as a reference blank. As NC, the latter two were not applied for maximal proteolysis. Finally, the intended zone was oversprayed with casein (2 μL/band, 20 mg/mL) to induce proteolysis. Additionally, trypsin and casein (volumes as mentioned) were individually applied as reference blanks. The plate was loaded with solutions (except for a band length of 8 mm and distance from the left edge of 18 mm), covered, wetted, incubated, and dried as described for HPTLC–nanoGIT (amylolysis inhibition)–FLD/Vis. After 10 min plate activation with a saturated solution of magnesium chloride (33% relative humidity) and 10 min plate preconditioning with the mobile phase, the plate was automatically developed with 10 mL 2-butanol/pyridine/ammonia (25%)/water (10:17:5:13, *V*/*V*/*V*/*V*) up to 50 mm and molecular sieve (0.3 nm) in the opposite trough (ADC2), taking 50 min all in all and dried afterward (120 °C, 10 min, TLC Plate Heater III). After derivatization of the peptides with the ninhydrin reagent as mentioned, the resulting chromatograms were detected at Vis (remission-transmission mode).

### 2.8 Spectrophotometric Trypsin Inhibition Assay for Comparison

For the L-BAPA trypsin inhibition assay,^22^ flour extract (non-purified, 3–300 μL) was mixed with trypsin (200 μL, 0.0135 mg/mL), filled to 800 μL with water, and preincubated at 37 °C for 10 min. The PC trypsin inhibitor (5–300 μL, 0.05 mg/mL) and NC (water) were used instead of the flour extract. The L-BAPA working solution (1 mL, 0.27 mg/mL) was added and incubated at 37 °C for 20 min. The reaction was stopped by adding 200 μL of aqueous acetic acid solution (30%). All samples were centrifuged at 17,000 *×* *g* for 3 min, and the absorbance of the released chromogenic product was measured at 410 nm using either a spectrophotometer (M501) or a combined hand-held cell counter and spectrometer (Fluidlab R-300). The final trypsin and L-BAPA concentrations in the 1.8 mL assay volume were 0.0015 and 0.15 mg/mL, respectively. The corresponding reference blanks were prepared without incubation and were immediately stopped by the addition of 200 μL acetic acid (30%).

### 2.9 Calculation of the Relative Inhibition

After the spectrophotometric α-amylase inhibition assay, the signal intensity (Int) of the flour extract subjected to the assay (assay) was corrected by the corresponding flour extract (blank) signal. The signal intensity (Int) of the NC was divided by this difference. This quotient was subtracted from 1 and multiplied by 100, as follows:

1 $$\mathrm{inhibition}\;\left(\%\right)=1-\frac{\mathrm{Int}\left(\mathrm{NC}\right)}{\mathrm{Int}\left(\mathrm{assay}\right)-\mathrm{Int}\left(\mathrm{blank}\right)}\times 100$$

After HPTLC–nanoGIT (amylolysis inhibition)–FLD/Vis, the corresponding signal intensity (Int) in the non-amylolyzed flour extract (blank) was subtracted from the saccharide signal in the flour extract subjected to the assay (assay) and divided by the signal intensity (Int) of the NC. This quotient was subtracted from 1 and multiplied by 100, as follows:

2 $$\mathrm{inhibition}\left(\%\right)=1-\frac{\mathrm{Int}\left(\mathrm{assay}\right)-\mathrm{Int}\left(\mathrm{blank}\right)}{\mathrm{Int}\left(\mathrm{NC}\right)}\times 100$$

After the spectrophotometric trypsin inhibition assay, the signal intensity of each flour extract subjected to the assay (assay) was corrected by the corresponding signal intensity of the flour extract (blank), divided by that of the NC, subtracted from 1, and multiplied by 100 as in eq 2.

## 3 Results and Discussion

### 3.1 Detection via Existing Spectrophotometric α-Amylase Inhibition Assays

The α-amylase inhibition potential can be detected either chemically via the iodine/iodide reagent^28^ reacting with the remaining starch or via the dinitrosalicylic acid reagent^21^ reacting with the formed saccharides, or biochemically via enzyme kits using chromogenic substrates releasing *p*-nitrophenol^29^ or fluorescent substrates.^25,13^ Among these, the iodine/iodide reagent and dinitrosalicylic acid reagent were tested as cuvette assays; however, the latter did not yield reproducible results due to the high flour extract matrix load of polysaccharides apart from the reducing saccharides reacting with the dinitrosalicylic acid reagent. The iodine/iodide reagent was thus the preferred choice, which does not assess the released products, but rather the decrease in the initial starch substrate via the absorption of the formed polyiodostarch complex. Exemplarily, the flour extract from refined wheat was subjected to a spectrophotometric α-amylase inhibition assay, in which inhibition of α-amylase resulted in more starch (amylolysis inhibition) and thus in higher absorption signal values compared to the NC (no inhibitor). The inhibition was calculated according to eq 1, and the inhibitor acarbose was used as the PC. Furthermore, a hand-held photometer (fluidlab R-300) was compared to a tabletop photometer (Camspec M501). For both spectrophotometric measurements (Table S1), acarbose inhibited amylolysis by 92%, and non-purified flour extract from refined wheat (300 μL) inhibited amylolysis by 71% and 75%, respectively. The extract strongly inhibited the α-amylase, which was almost as strong as that of acarbose, confirming the presence of inhibitors. Thus, the extract was suited to develop a quantitative HPTLC–nanoGIT (amylolysis inhibition)–FLD/Vis method as follows.

### 3.2 Development of HPTLC–NanoGIT (Amylolysis Inhibition)–FLD/Vis

Our previous approach combined the in-vial preincubation of α-amylase with ATI-containing flour extract and the subsequent on-surface incubation with starch as a substrate.^26^ Here, it was aimed to perform the complete workflow on the same surface in one step to make the whole analysis faster and more efficient. Therefore, the incubation period for amylolysis was shortened, the on-surface incubation process was streamlined, and for detection, a derivatization reagent sequence was used. For the enzyme–substrate reaction (amylolysis), the incubation time was reduced from 60 to 10 min (Figure S1). An initial separate on-surface preincubation of α-amylase with the flour extract (30 min), followed by the enzyme–substrate reaction (10 min), led to signal loss as the plate was wetted twice (Figure S1). The plate drying between both incubation steps (15 min, cold stream of air, hair dryer) was insufficient, and the residual humidity on the plate led to zone diffusion. Therefore, to streamline the on-surface incubation process, a single incubation step was performed, in which the inhibitor, enzyme, and substrate were incubated together for 30 min, ensuring adequate interaction (Figure S2). A reagent sequence on the same plate proved advantageous for detection, as amylolysis resulted in less glucose (Glc) than maltose (Mal) and maltotriose (Mal3).^27^ The *p*-aminobenzoic acid reagent detected monosaccharides, such as Glc, up to a factor of 13 more sensitive,^30^ whereas the 2-naphthol reagent detected all saccharides to a similar extent. Successful inhibition by flour extract from refined wheat (non-purified) was observed for the released Mal3. After dialysis, the remaining polysaccharides in the flour extract^26^ (retained in the start zone besides starch) were cometabolized, leading to an increase in Mal, whereas no change in intensity was apparent for Glc. Hence, the inhibition of amylolysis in a single incubation step was successfully demonstrated.

Our previous observation^26^ was confirmed that the PC acarbose (a tetra-pseudosaccharide) did not only inhibit the α-amylase but was also metabolized by the α-amylase (Figure 1). When α-amylase was incubated with acarbose, the Glc and Mal zones increased by 29% and the Mal3 zone increased by 17% compared to the respective saccharide signals for only α-amylase (Table S2). However, with increasing amounts of acarbose (Figure 1, 1–5 μg/band) and thus increasing inhibition of on-surface amylolysis, the release of Glc (−1 to −24%), Mal (−5 to −26%), and Mal3 (−6 to −24%) was visibly reduced. The Mal3 release was difficult to evaluate with the *p*-aminobenzoic acid reagent because of its weak signal intensity and zone diffusion.

**Figure 1:**
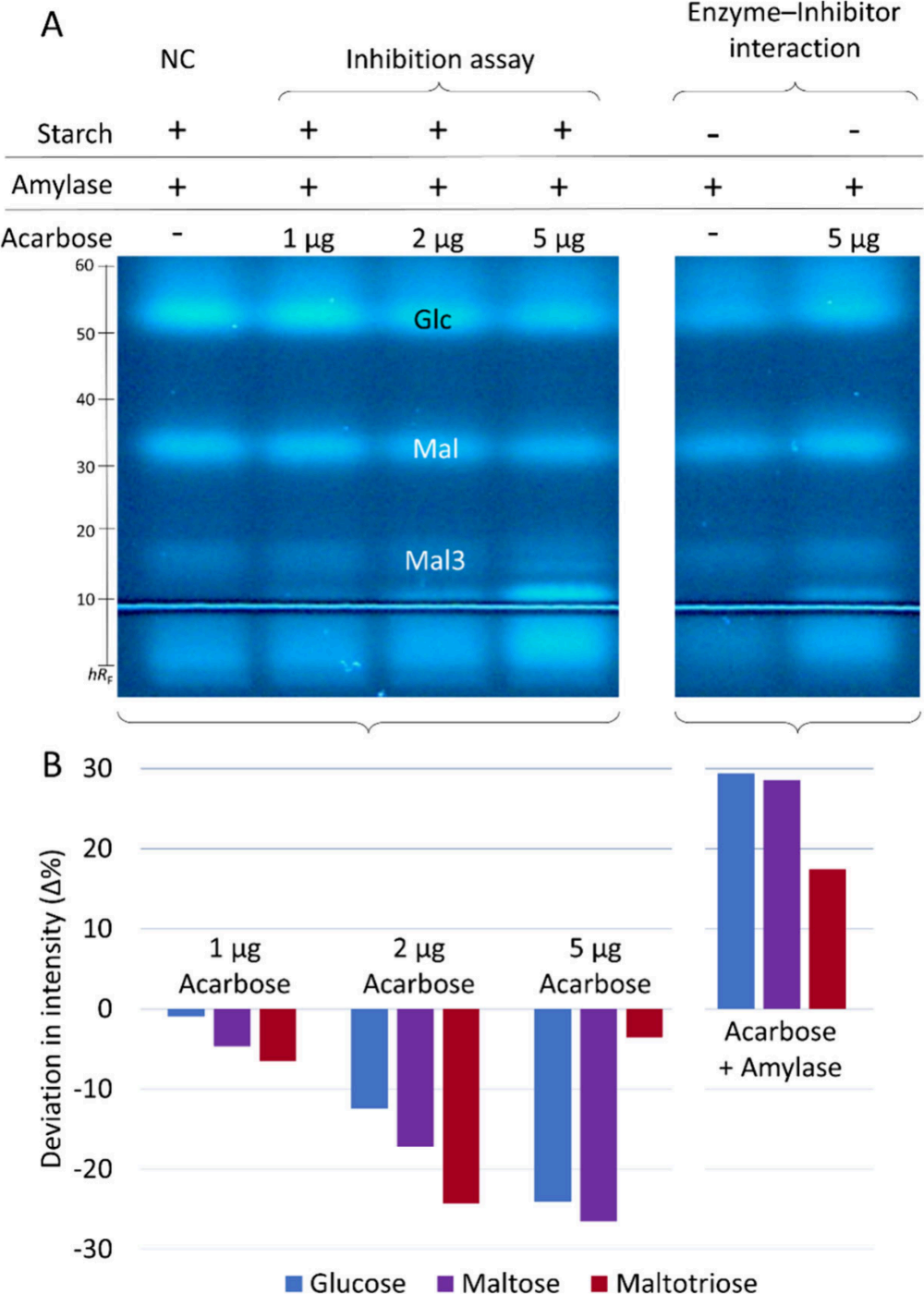
Inhibition of amylolysis by different acarbose amounts (1–5 μg/band): (A) HPTLC–nanoGIT (amylolysis inhibition)–FLD chromatogram and (B) percentage deviation in intensity (Δ%) of released maltotriose (**Mal3**), maltose (**Mal**), and glucose (**Glc**) in contrast to the NC (α-amylase, 2.5 μg/band and soluble starch, 4 μg/band) and for acarbose (2 μg/band) oversprayed by α-amylase (2.5 μg/band) in contrast to α-amylase (2.5 μg/band); analyzed on HPTLC plate silica gel 60 with acetonitrile/water/2-propanol/acetone (12:3:4:1, *V*/*V*/*V*/*V*) up to 70 mm, derivatized with *p*-aminobenzoic acid reagent, detected at FLD 366 nm, and quantified after densitometric fluorescence measurement at 366/>400 nm.

The highly sensitive detection of saccharides clearly revealed that the α-amylase was massively impurified with Glc, Mal, and Mal3 (Figure 1). Our previous removal of saccharides using a 3k-Da Amicon Ultra filter still showed residual mono- and oligosaccharides in the retentate of flour extracts and α-amylase,^26^ but filters with a higher retentate volume were not available. Therefore, another type of membrane filtration was carried out using a D-Tube Dialyser Mega (MWCO 3.5 kDa). Although it took longer (12 h instead of 2 h) to remove the mono- and oligosaccharides (permeate), there was no sample concentration or clogged membrane hindering saccharide removal. Different periods were tested, and a 12 h dialysis with salt load renewal after every two cycles (every 4 h) was suited to remove saccharides from α-amylase (Figure 2, marked −). After a 2 h dialysis, the Mal3 zone was neither detectable with the *p*-aminobenzoic acid reagent nor the 2-naphthol reagent. The intensity of the Mal zone (dark blue curve) decreased more with increasing dialysis time than that of the Glc zone (orange curve), which was best detectable with the 2-naphthol reagent and *p*-aminobenzoic acid reagent, respectively (Figure 2). The Glc zone was still clearly detectable after a 12 h dialysis, whereas Mal and Mal3 were satisfactorily removed (Figure 2).

**Figure 2:**
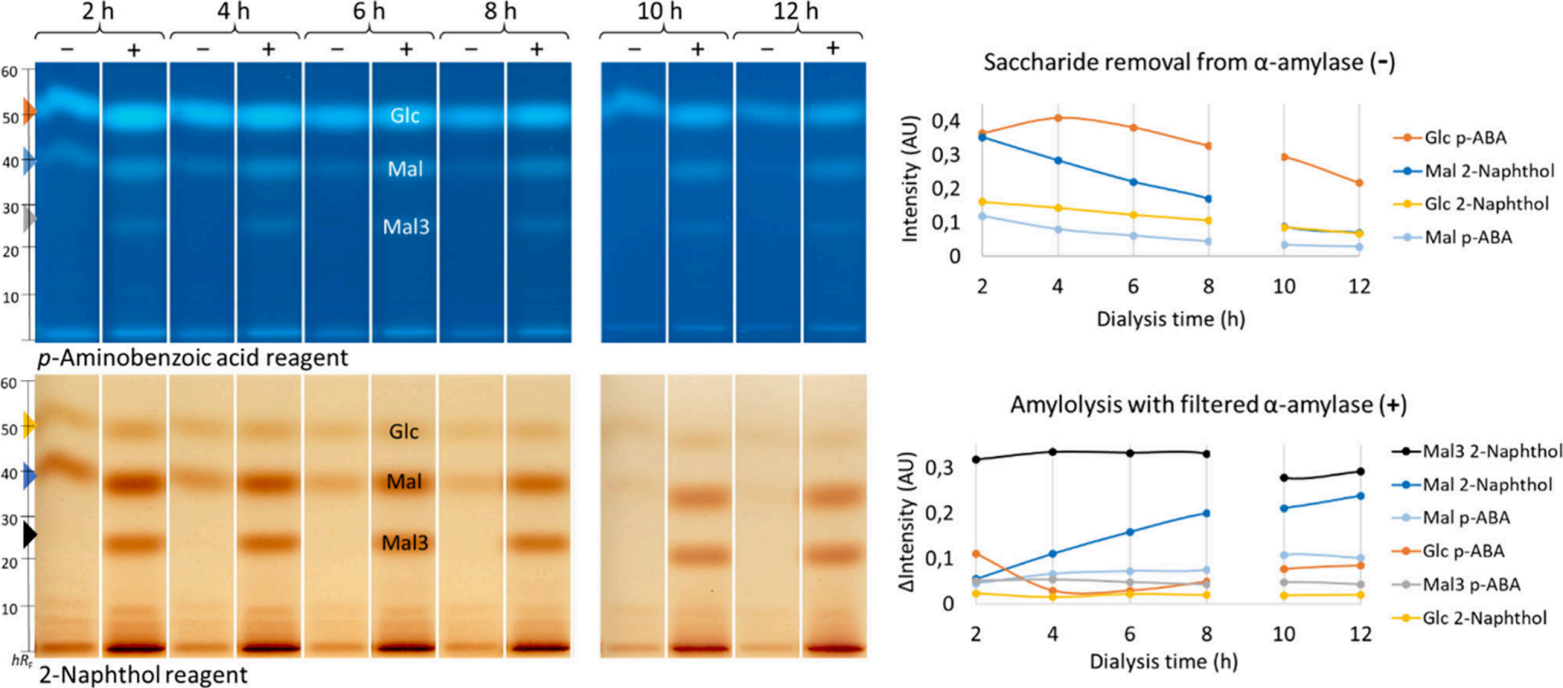
Saccharide removal from α-amylase (5 mg/mL) over a 2–12 h dialysis period: HPTLC–nanoGIT (amylolysis inhibition)–FLD/Vis chromatograms (2–8 h on plate 1 and 10-12 h on plate 2) and respective graphs of filtered α-amylase (2.5 μg/band, −) overspotted with soluble starch (4.0 μg/band, +) versus amylolysis to prove the activity of the purified α-amylase; analyzed on HPTLC plate silica gel 60 with acetonitrile/water/2-propanol 3:1:1 (*V*/*V*/*V*) up to 70 mm, derivatized via a reagent sequence first with the *p*-aminobenzoic acid reagent (*p*-ABA), detected at FLD 366 nm, and quantified after densitometric fluorescence measurement at 366/>400 nm, followed (on the same plate) by the 2-naphthol reagent, detected at Vis (remission-transmission mode), and quantified after densitometric absorbance measurement at 500 nm.

To ensure proper enzymatic activity of the purified α-amylase, on-surface amylolysis was performed (Figure 2, marked +), and the zone intensities of the released saccharides, corrected by the saccharides retained in the purified α-amylase, were compared with each other (Figure 2). No differences in enzymatic activity were observed over the 12 h period, although Mal and Mal3 being sensitively detected using the 2-naphthol reagent, and Glc using the *p*-aminobenzoic acid reagent. Surprisingly, the saccharide release of Mal via the 2-naphthol reagent (dark blue curve) increased constantly over the 12 h period, which was not observed to this extent via the *p*-aminobenzoic acid reagent (light blue curve). The lower the amount of Mal present in the sample, the more Mal was released during amylolysis. If Mal acts as a putative inhibitor of amylolysis,^31–33^ its removal could cause an increase in activity and, thus, higher Mal release; however, no increase in other saccharides was observed. Additionally, the 10 h and 12 h dialyzed α-amylase were not applied onto the same HPTLC plate, and thus, were not derivatized together with the ones collected after 2–8 h, so these intensity signals must be compared with caution, which is why only the delta-intensity (amylolysis (+) tracks subtracted by amylase (−) tracks) was compared. The Glc intensity via the *p*-aminobenzoic acid reagent (orange curve) decreased after 4 h dialysis and then slightly increased, which could be due to minor fluctuations in the random enzymatic reaction because the activity was constant via 2-naphthol derivatization (yellow curve), which, however, was not as sensitive to short-chain saccharides.

As mentioned, native polysaccharides in the flour extract, which were co-metabolized in addition to starch by α-amylase, led to an increase in the Mal zone after inhibition of amylolysis by these (Figure S2), which is why the flour extracts were also dialyzed. The flour extract from whole wheat was selected to evaluate the dialysis time required due to its expected high contents of Glc, Mal, and Mal3 (Figure S3). A 12-h period with salt load renewal after every two cycles (every 4 h) was sufficient to remove most of the saccharides. Analogously, the refined wheat and einkorn flour extracts were dialyzed for 12 h and used as the corresponding flour extract blanks.

### 3.3 Quantification of Amylolysis Inhibition by ATI-Containing Flour Extracts Using HPTLC–NanoGIT (Amylolysis Inhibition)–FLD/Vis

After the successful development of the all-in-one HPTLC–nanoGIT (amylolysis inhibition)–FLD/Vis method, inhibition by ATI-containing purified flour extracts from refined wheat, whole wheat, and einkorn was investigated (Figure 3). Two derivatization reagents were used as reagent sequence (first *p*-aminobenzoic acid reagent and then 2-naphthol reagent) and compared to determine the best detection mode. The PC acarbose was used for comparison (Table S3). As mentioned, the detectability of the released saccharides was different for the two reagents. The difference between both reagents was highest for Glc (16–29%), followed by Mal3 (13–17%), and Mal (3–8%).

**Figure 3:**
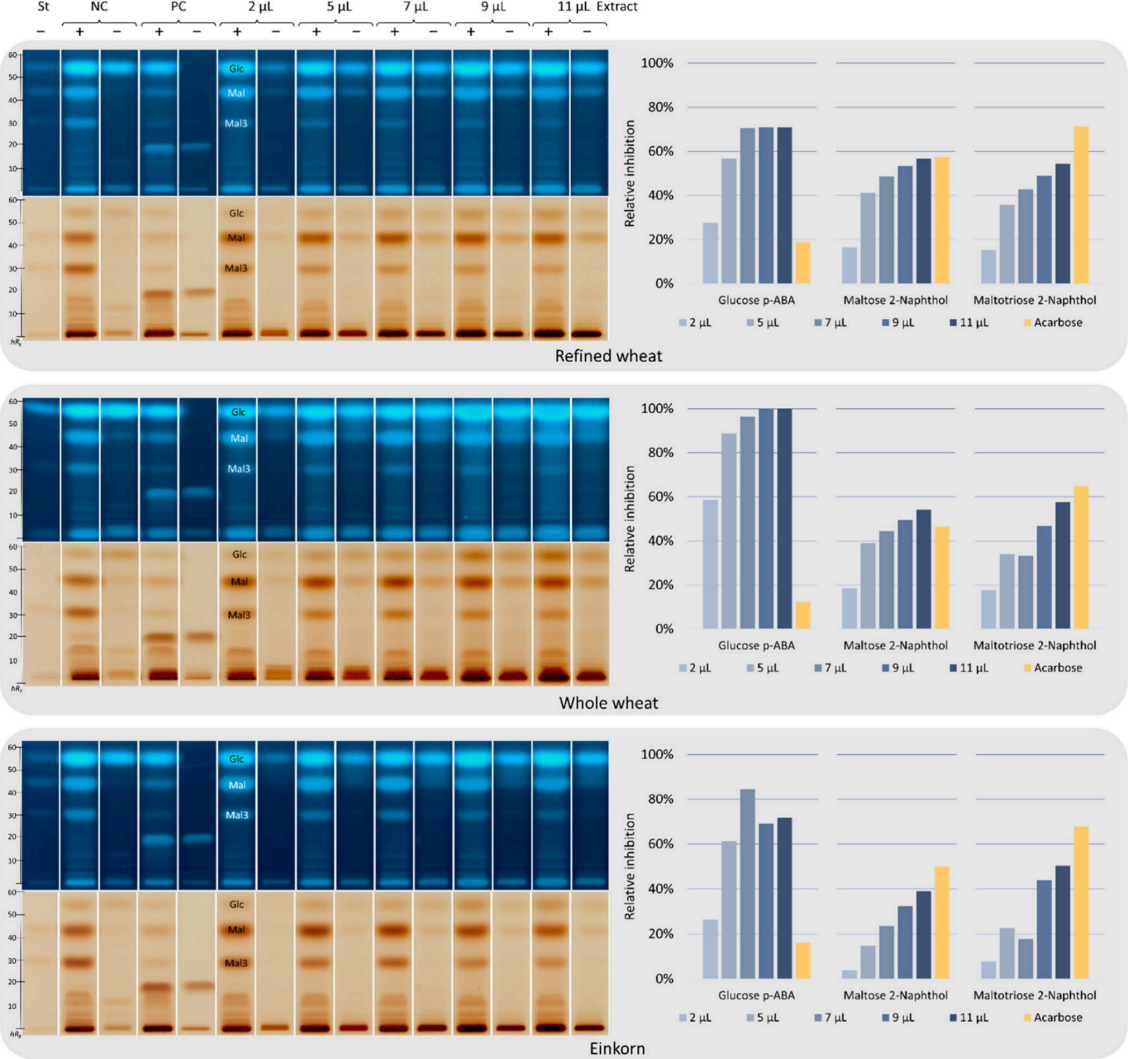
Quantitative all-in-one amylase inhibition assay: HPTLC–nanoGIT (amylolysis inhibition)–FLD/Vis chromatograms (*n* = 1), developed, derivatized, and analyzed as in Figure 2, showing different volumes (2–11 μL/band) of 12-h dialyzed flour extracts from refined wheat, whole wheat, and einkorn or **NC** (no flour ATI extract applied) or **PC** acarbose (5 μg/band) oversprayed with 12-h dialyzed α-amylase (25 μg/band) and soluble starch (20 μg/band) for amylolysis (+) versus corresponding non-amylolysed (−) counterparts and standards (**St**; **Glc** 0.5 μg/band, **Mal** and **Mal3** 1 μg/band).

For 3-fold analysis on three different plates, Mal3 (2% and 5%) showed the best and Glc the worst (21% and 23%) interday precisions for the *p*-aminobenzoic acid reagent and 2-naphthol reagent, respectively (Table S3). The *p*-aminobenzoic acid reagent was slightly more precise for all saccharides than the 2-naphthol reagent (2–21% versus 5–23%), and the calculated relative inhibition by acarbose was lower via Glc release and higher via Mal and Mal3 release. In particular, the detection of Glc was challenging, as it was close to the LOD/LOQ. A reduction/increase in Glc release alone was not possible because it was limited by amylolysis, releasing all products in specified amounts. The precision values were only worse for those saccharides (Glc and Mal), for which α-amylase was impure. However, the detectability of Glc was better with the *p*-aminobenzoic acid reagent, and of Mal and Mal3 with the 2-naphthol reagent; the inhibition by the flour extracts was evaluated for this detection combination (Figure 3, all in Tables S4–S6).

As expected, the relative inhibition of amylolysis (calculated via the reduced release of each saccharide, eq 2) increased continuously with increasing volume of all ATI-containing flour extracts, except for Glc from the einkorn extract, with a maximum of 85% at 7 μL. The decrease in the relative inhibition at higher volumes could be explained by an inhibitor which tends toward side reactions (dissociation of enzyme–inhibitor complex or creation of inhibitor–substrate or inhibitor–inhibitor complexes)^34^ and thus becomes unavailable for further inhibition. This effect was also observed in the spectrophotometric trypsin inhibition assay (section 2.2.2), where only einkorn showed a decrease in relative inhibition at higher volumes. Inhibition by flour extracts from refined and whole wheat, with respect to Glc, reached saturation at approximately 70% and 100%, respectively. The relative inhibition of nearly 100% was difficult to evaluate due to the high native amount of Glc in whole wheat. Subtracting this amount, the corrected intensity signal was negative, resulting in inhibition of 100% and 104% for the 9 μL and 11 μL flour extracts, respectively. Comparing the flour extracts from refined and whole wheat, the latter had a higher relative inhibition (based on Glc), but also showed the highest native Glc amount. The reduction in released Mal and Mal3 was slightly higher for the flour extract from refined wheat. Because ATIs are present in the endosperm,^35,36^ a higher inhibition by the flour extract from refined wheat was assumed. However, the reduced release of Mal and Mal3 indicated a similar α-amylase inhibition. The flour extract from einkorn inhibited α-amylase to a lesser extent than that of refined wheat. This confirmed previous hypotheses.^17,29^ Also, another study found a correlation between ATI content of different cereals and α-amylase inhibition was not evident, indicating the presence of other inhibitors as well as reduced inhibition of human salivary α-amylase.^25^ The developed HPTLC approach has the potential for detailed analysis of the linkage between inhibitor identification and inhibition potential, when combined with other identification techniques such as mass spectrometry.

Quantification of the inhibition of α-amylase by ATI-containing flour extracts was possible via HPTLC–nanoGIT (amylolysis inhibition)–FLD/Vis. The einkorn extract showed lower inhibition than the refined and whole wheat, whereas the latter showed no difference. In contrast to our previous study,^26^ better purification of flour extracts resulted in more differentiated results. Since purification of flour and α-amylase is preferable to subtraction of the respective blank signal intensity, particularly when close to the detection limit (2-naphthol reagent) or signal saturation/detector limit (*p*-aminobenzoic acid reagent), the extracts will need to be further purified in the future. Furthermore, different origins of α-amylase^27^ should be investigated to clarify their affinities.

### 3.4 Spectrophotometric Trypsin Inhibition Assay

For comparison, a spectrophotometric trypsin inhibition assay was performed with L-BAPA as a substrate,^22^ and the inhibition was calculated according to eq 2. Again, the tabletop photometer was compared with the hand-held photometer and showed no difference in relative inhibition (Figure S4, Table S7). The first experiment, which yielded no product release, showed that L-BAPA was not interchangeable with DL-BAPA, as suppliers had suggested. Trypsin inhibitor from *Glycine max* was chosen as PC, and different final concentrations (0.03–8.33 μg/mL) were used to successfully prove a linear inhibition range (Figure S4A, Table S7). Since a linear increase in inhibition followed by a subsequent decrease at higher flour extract volumes has been reported,^22^ trypsin inhibition was investigated for increasing volumes (3–300 μL) of the flour extracts (Figure 4, Table S7). At higher volumes (>80 μL), a decrease at almost 100% inhibition was observed only for einkorn, as already discussed for HPTLC–nanoGIT (amylolysis inhibition)–FLD/Vis. The flour extracts from refined and whole wheat showed a nearly linear increase up to 100 μL and then reached a plateau at 100% inhibition, contrary to the results of a previous study.^22^ The curve and inhibition potential were very similar for refined versus whole wheat. An inhibition of nearly 100% for all three cereal types was observed at high extract volumes. Surprisingly, the assay reproducibly resulted in negative inhibition (increasing trypsin activity) at low extract volumes (3–15 μL) for all three types of cereals. This phenomenon has not been reported to date, and only assumptions regarding the increase in trypsin activity can be made. The flour ATI-containing extract either contained an activity-increasing cofactor/enzyme, which was suppressed/compensated at higher inhibitor concentrations, or an L-BAPA-destabilizing agent, which suggests higher enzyme activity.

**Figure 4:**
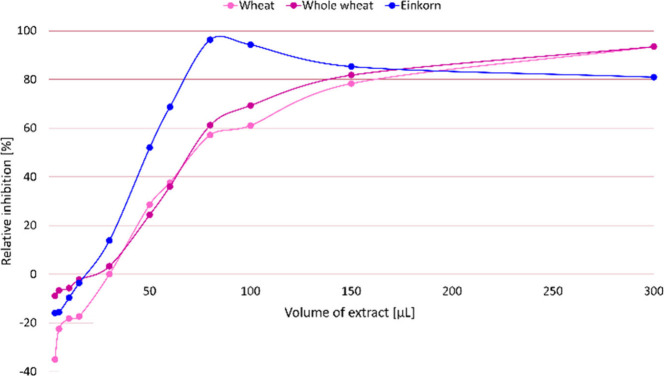
Mean relative inhibition (via tabletop and hand-held spectrophotometer, individual relative inhibitions in Figure S4 and data sets in Table S7, *n* = 2) of trypsin by flour extracts from refined wheat, whole wheat, and einkorn (3–300 μL) in the spectrophotometric trypsin inhibition assay (1.8 mL assay volume, 0.0015 mg/mL trypsin, and 0.15 mg/mL L-BAPA).

### 3.5 In-Vial Trypsin Inhibition Assay, Followed by HPTLC Separation

The in-vial trypsin inhibition assay followed by HPTLC separation was performed on wettable reversed-phase HPTLC plates.^26^ After several unsuccessful trials of on-surface proteolysis on this batch-dependent plate (data not shown), the plate type was changed to a more batch-robust and cheaper normal-phase plate. First, the mobile phase for peptide separation (19.5:17:5:13, *V*/*V*/*V*/*V)* ^37^ was optimized to ensure good selectivity. The best separation was achieved by 2-butanol/pyridine/ammonia (25%)/water 10:17:5:13 (*V*/*V*/*V*/*V*), in which 2-butanol was halved. Before the implementation of the on-surface assay, the optimal enzyme–inhibitor ratio (E/I) was evaluated via an in-vial trypsin inhibition assay using a trypsin inhibitor from *Glycine max* (TI) as PC and the protein casein as the substrate. An abrupt strong inhibition was observed between an E/I of 1:2 and 1:3 (Figure S5), which could point to competitive inhibition.^34^ To differentiate false positive results for the highest E/I from a failed assay, both E/I ratios were analyzed in the following.

First, it was excluded that the protein-containing flour extract was digested by trypsin. The peptides detected for trypsin mixed with flour extract from refined wheat (no casein present) were assigned to the matrix signals from the extract (Figure S6). The optimal incubation period and volume of the flour extracts were also evaluated using an in-vial trypsin inhibition assay (Figure S6). No difference was observed for different preincubation times (10–30 min) of the enzyme and inhibitor, and an increase in inhibition of proteolysis by flour extract from refined wheat was only visible between 100 and 150 μL, whereas a further increase in the volume (175 μL) did not increase the inhibition.

Thus, a preincubation time of 10 min, an E/I ratio of 1:2 and 1:3 for trypsin inhibitor, and flour extract volumes of 100–150 μL were selected for further analysis. The three different types of flours were compared using the in-vial trypsin inhibition assay (Figure 5) with respect to any difference between whole grain and refined flours,^35,36^ and einkorn as a reported ATI-poor cereal.^38,17,36^ For distinct peptide signals (Figure 5, framed), inhibition was observed. The flour extract from refined wheat slightly inhibited proteolysis (marked +) compared to that of whole wheat, whereas the evaluation of einkorn was difficult due to high matrix loads. In particular, strong blank signals (marked −) were detected for the flour extract from einkorn. A comparison of the PC and flour extracts showed that not the same peptides were inhibited to the same extent, which was most probably caused by different active trypsin centers. With increasing amounts of PC, a clear increase in inhibition was evident (Figure 5), whereas in the repetition of the analysis on the second plate, both E/I showed complete inhibition (Figure S7). In addition, the NC showed different intensities of the peptide signals on both plates, indicating that the stronger inhibition by the PC could be overlaid by an overall lower signal intensity on the second plate (Figure S7). It was found that the separation was susceptible to relative humidity, which was controlled to be approximately 33% on the plate surface by preconditioning with saturated magnesium chloride solution and as dry as possible (<15%) during development using a molecular sieve (0.3 nm) in further analyses.

**Figure 5:**
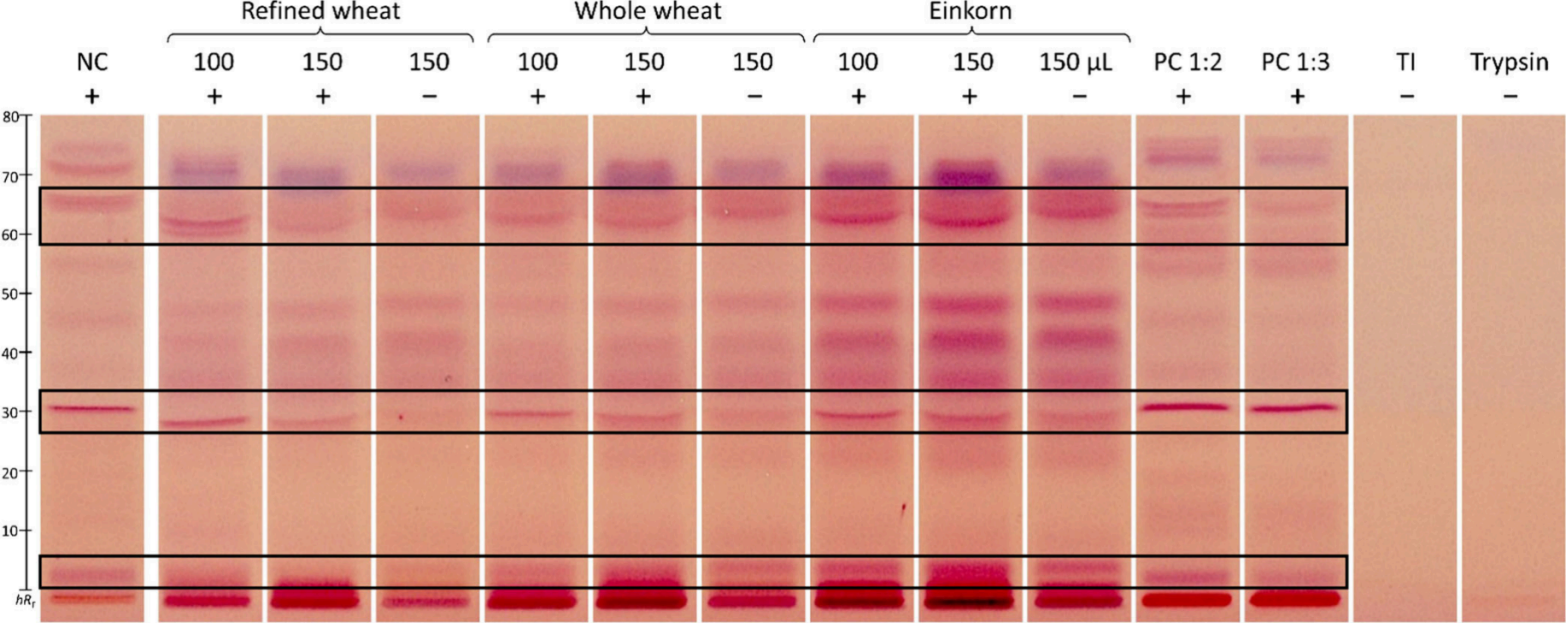
In-vial trypsin inhibition assay (7 μL/band), followed by HPTLC analysis: HPTLC–Vis chromatograms showing inhibition of proteolysis (marked +, framed region of interest) by three flour extracts (100 and 150 μL each) versus corresponding non-proteolyzed flour extracts (150 μL, marked −), proteolyzed casein (**NC**, 1:100), proteolyzed trypsin inhibitor (**PC 1:2** and **1:3**, mixed with trypsin 1:2 and 1:3 in a vial, 10 min preincubated, and 30 min incubated at 37 °C), TI, and trypsin; analyzed on HPTLC plate silica gel 60 with 2-butanol/pyridine/ammonia (25%)/water 10:17:5:13 (*V*/*V*/*V*/*V*) up to 50 mm, derivatized with ninhydrin reagent, and detected at Vis (remission–transmission mode).

### 3.6 Development of the On-Surface Proteolysis Inhibition

For full on-surface proteolytic inhibition, the optimal on-surface enzyme–substrate ratio (E/S) was evaluated (Figure S8). Unfortunately, casein showed weak peptide signals in the absence of trypsin. This was explained by the higher absolute amounts analyzed, i.e., a 50–100-fold higher amount of casein via the on-surface approach (40–80 μg/band) than in the in-vial assay (0.8 μg/band). Standard enzyme assay protocols usually only specify the E/S, so that each user can adapt these ratios to specific applications. However, the evaluation of different absolute amounts of trypsin and casein, but still the same E/S, revealed strong differences in the peptide signals. Nevertheless, the higher the amount of trypsin and casein, the stronger the peptide signals in comparison with casein blanks which proved successful on-surface proteolysis (Figure S8a–d). The casein solution was a difficult substrate due to the presence of casein micelles, which is why highly concentrated solutions were needed to achieve higher amounts with lower volumes. This led to the partial destruction of the plate at the application zone which impaired the separation (Figure S8d). Thus, 40 μg/band casein (2 μL/band, 20 mg/mL) was the maximum amount that could be applied satisfactorily. After plate drying (cold stream of air, hair dryer), the separation of peptides was prone to residual humidity, resulting from the incubation of the plate. In contrast, plate heating (120 °C, 10 min) was more effective. It did not influence the amount of peptide signals (Figure S8) or lead to denaturation of proteins or false-positive peptide signals. Although an E/S of 1:100 has been recommended,^22^ the best peptide signals were obtained at a lower E/S of 1:50. The order of the solutions applied/oversprayed on the same start zone was as follows. First, trypsin was applied, followed by the inhibitor, and finally the substrate.

### 3.7 Comparative Inhibition of the Proteolysis by ATI-Containing Flour Extracts via HPTLC–NanoGIT (Proteolysis Inhibition)–Vis

Using the developed HPTLC–nanoGIT (proteolysis inhibition)–Vis, the three flour extracts and PC (TI) were applied on the HPTLC plate via overspotting and incubated for 30 min to ensure optimal interactions (Figure 6). The plate layout did not provide enough space for both E/I ratios of 1:2 and 1:3; thus, an E/I of 1:2.5 was chosen. The PC (E/I 1:2.5) almost completely inhibited proteolysis, and only weak peptide signals were still visible, confirming successful on-surface proteolysis inhibition. Due to the strong native peptide signals of the flour extracts at volumes of 4 and 7 μL (Figure 6), an additional volume of 2 μL was chosen, and inhibition of proteolysis was still observed (Figure S9). A comparison of different cereal flour extracts and volumes revealed only slight differences in inhibition, except for einkorn at higher volumes (Figure 6, framed). The flour extract from einkorn showed stronger inhibition of some peptide signals than the refined and whole wheat extracts, which was in accordance with the literature.^29^ ATI-containing flour extracts from refined and whole wheat had a similar trypsin inhibitory potential (no difference was evident).

**Figure 6:**
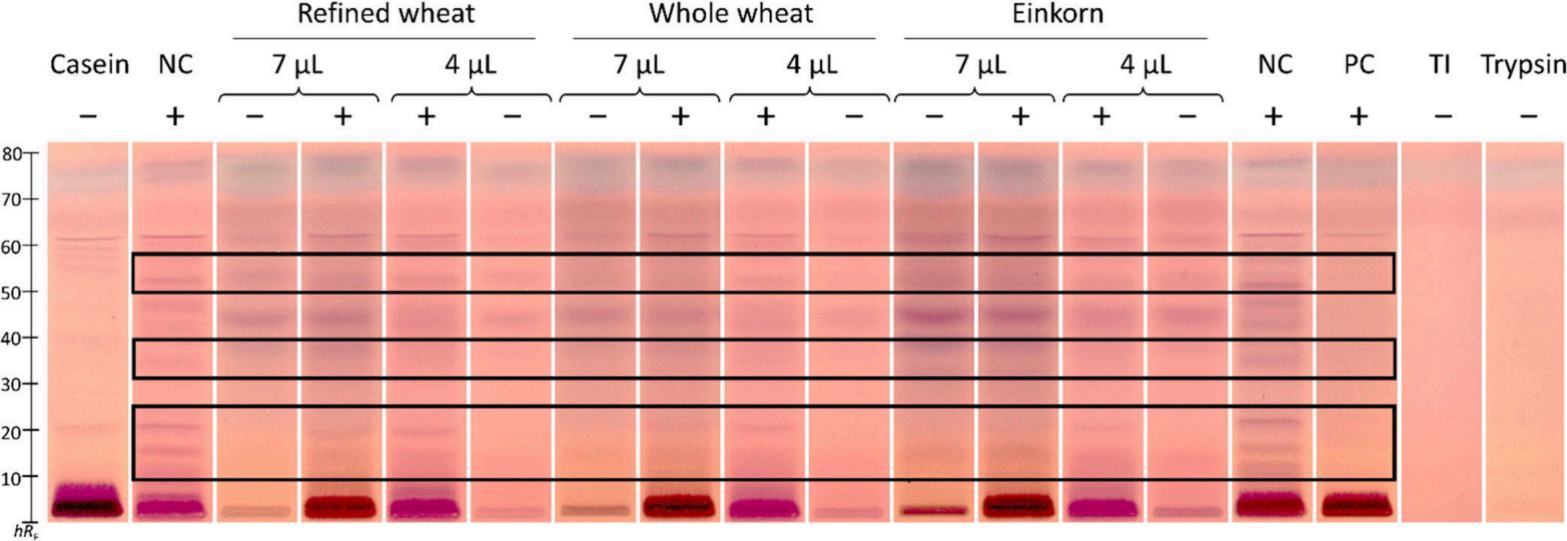
Qualitative all-in-one trypsin inhibition assay: HPTLC–nanoGIT (proteolysis inhibition)–Vis chromatograms showing the inhibition of the proteolysis (marked +, framed region of interest) by three flour extracts (4 and 7 μL/band) versus corresponding non-proteolyzed flour extracts (marked −) and casein (2 μL/band, 20 mg/mL), maximal proteolysis (**NC**, 2-fold determination, casein/trypsin E/S 1:50, 30 min incubation), maximal inhibition (**PC**, trypsin/trypsin inhibitor E/I 1:2.5) versus trypsin inhibitor (**TI**, 1 μL/band, 0.5 mg/mL), and trypsin (4 μL/band, 0.2 mg/mL); analyzed on HPTLC plate silica gel 60 with 2-butanol/pyridine/ammonia (25%)/water 10:17:5:13 (*V*/*V*/*V*/*V*) up to 50 mm, derivatized with the ninhydrin reagent, and detected at Vis (remission–transmission mode).

Although the native peptides in the extracts hindered the easy evaluation of the trypsin inhibition potential, the on-surface assay can still be used to study the inhibition process. Again, dialysis of flour extracts with a higher or even the same molecular weight cut-off as used here could exclude small peptide signals without losing the ATIs. Because it could not be excluded that the flour extracts contained other inhibiting compounds besides the ATIs, prefractionation of the extract via HPLC^25^ and analysis of the fractions via the developed on-surface method could identify causative substances and reduce matrix signals.

### 3.8 Comparison of Methodologies

Both hypotheses turned out to be true for the HPTLC–nanoGIT amylolysis inhibition workflow, which was performed entirely on the surface (in one step) and quantitatively. For refined wheat flour extract, the workflow was compared to the conventional spectrophotometric α-amylase inhibition assay. Despite differences in enzyme–inhibitor ratios (1:150 for the spectrophotometric versus 1:44 for the HPTLC approach), both methods produced comparable inhibition trends. The HPTLC approach required considerably less substrate (2.5-fold) and inhibitor (3.4-fold) and showed 62% α-amylase inhibition, making it more efficient, while still providing detailed information on the individual saccharides released during amylolysis. This was only 11% less than the inhibition calculated via the spectrophotometric approach.

Overall, the HPTLC–nanoGIT workflow not only confirmed the inhibitory effects observed with the spectrophotometric assay but also provided substantial resolution of saccharide-specific interactions, which can not be reached with the spectrophotometric assay or is difficult to achieve with conventional methods. Since both approaches used starch as a substrate, better comparability could be achieved by reducing the E/I and E/S of the spectrophotometric approach bearing in mind the limit of detection of the iodine/iodide reagent. Nevertheless, the developed HPTLC–nanoGIT (amylolysis inhibition)–FLD/Vis allowed clear and differentiated results regarding the single saccharides released during amylolysis and their interactions with α-amylase.

The first hypothesis turned out to be true for the HPTLC–nanoGIT proteolysis inhibition workflow performed in one step, entirely on the surface. The second hypothesis was not fulfilled: The HPTLC–nanoGIT proteolysis inhibition workflow allowed a comparative evaluation of the inhibition of proteolysis by different ATI-containing flour extracts but was not suitable for quantification because of the dominant peptide signals in the blank samples. The spectrophotometric trypsin inhibition assay showed nearly 100% inhibition for the three cereal types at higher extract volumes (300 μL), whereas einkorn showed maximum inhibition already at 100 μL. Comparability was reached for an E/I of 1:0.7 to 1:66 and E/S of 1:100 for the spectrophotometric trypsin inhibition assay and respectively an E/I of 1:0.6 to 1:2 and E/S of 1:50 for HPTLC–nanoGIT (proteolysis inhibition)–Vis. Hence, in the overlapping E/I ratio of 1:0.6 to 1:2, flour extract volumes were compared (3–100 μL versus 2–7 μL). In this E/I range, the curve for the spectrophotometric trypsin inhibition assay (Figure 4) was linear, and thus comparable evaluation was legitimate. At the highest E/I, the inhibition by flour extracts from refined wheat, whole wheat, and refined einkorn was 60%, 70%, and 100%, respectively, and was negative at the lowest E/I. For HPTLC–nanoGIT (proteolysis inhibition)–Vis, a difference of 10% in inhibition between refined and whole wheat was difficult to detect. The chromatograms revealed total trypsin inhibition by flour extract from einkorn was not observed for any of the peptide signals, whereas the spectrophotometric trypsin inhibition assay showed 100% inhibition for all three types of cereal at higher extract concentrations. Concluding, 100% inhibition could only be partially confirmed to a limited extent with the on-surface HPTLC trypsin inhibition assay, where further reduction of the matrix peptide signals is helpful. Nevertheless, matrix reduction is also preferred for spectrophotometric assays to prevent negative relative inhibition values. However, the comparison of immobilized on-surface assays and liquid-based spectrophotometric approaches is hard to realize because of the very different environmental assay conditions. Thus, the E/I and E/S ratio is very important when comparing different levels of platforms; additionally, it is helpful to calculate a factor of difference between methodologies if quantification was achieved. A method validation is always preferable to ensure comparability.

In conclusion, for the first time, both developed on-surface methods were established as reliable, even superior alternatives to existing spectrophotometric inhibitor assays, and can be used complementary to better estimate the inhibition by ATIs. In contrast to mass spectrometric approaches, the HPTLC–nanoGIT methods could be used to identify not only known but also unidentified inhibitors in combination with further purification and effect-directed analysis. The power of both HPTLC–nanoGIT methods is evident, as they provided a profile pattern of the released individual metabolisation products and compared these for different inhibitors, thus identifying different modes of inhibition for different inhibitors. Comparatively, more information was obtained, which is helpful for understanding the underlying mechanisms important for further research about NCWS, its triggers, and enzyme interactions. For an affordable start, the inexpensive 2LabsToGo system^39–41^ can be used to apply the comparatively more informative and sustainable on-surface methodology.

## Supplementary Material



## Notes

Raw data from the on-surface and spectrophotometric inhibition assays and additional figures of the method optimization (PDF)
