# Supplementary material for: Potential of Quantitative α‑Amylase or Trypsin Inhibition by Refined and Whole Wheat and Einkorn Using High-Performance Thin-Layer Chromatography–NanoGIT versus Conventional Spectrophotometry
Source: J Agric Food Chem. 2026 Feb 20;74(8):7221–32. doi: 10.1021/acs.jafc.5c14009 (PMC12964541; doi:10.1021/acs.jafc.5c14009)
Supplement: Supplementary file 1 [file jf5c14009_si_001.pdf]

Supplementary information

Potential of quantitative α-amylase or trypsin inhibition by refined and whole wheat and einkorn using high-performance thin-layer chromatography–nanoGIT versus conventional spectrophotometry

Isabel Müller, Ilka Scheibelhut, Gertrud E. Morlock*

Chair of Food Science, Institute of Nutritional Science, and Interdisciplinary Research Centre for Biosystems, Land Use and Nutrition, Justus Liebig University Giessen, Heinrich-Buff-Ring 26-32, 35392 Giessen, Germany

*Corresponding authors: Prof. Dr. Gertrud Morlock, phone: +49-641-9939141; fax +49-641-99-39149, email: gertrud.morlock@uni-giessen.de

Table of contents

**Table S1**	Spectrophotometric α-amylase inhibition assay of wheat flour extract and acarbose as positive control evaluated by the handheld anvajo fluidlab and a tabletop photometer (Camspec M501).	S5
**Table S2**	Evaluation of the on-surface α-amylase inhibition by acarbose and its metabolism by α-amylase for each saccharide described as the percentage deviation (Δ%) from amylolysis and α-amylase, respectively.	S6
**Table S3**	α-Amylase inhibition by acarbose in each HPTLC–nanoGIT (amylolysis inhibition)–FLD/Vis analysis and the difference between the derivatization reagents *p*-aminobenzoic acid and 2-naphthol and the determined precision (n = 3) as relative standard deviation (RSD) for each saccharide.	S7
**Table S4**	Determined signal height of each saccharide of the HPTLC–nanoGIT (amylolysis inhibition)–FLD/Vis analysis of refined wheat, corrected signal, and calculated relative inhibition of both derivatisation reagents.	S8
**Table S5**	Determined signal height of each saccharide of the HPTLC–nanoGIT (amylolysis inhibition)–FLD/Vis analysis of whole wheat, corrected signal, and calculated relative inhibition of both derivatisation reagents.	S11
**Table S6**	Determined signal height of each saccharide of the HPTLC–nanoGIT (amylolysis inhibition)–FLD/Vis analysis of einkorn, corrected signal, and calculated relative inhibition of both derivatisation reagents.	S14
**Table S7**	Spectrophotometric trypsin inhibition assay with trypsin inhibitor and flour extracts of refined wheat, whole wheat, and einkorn.	S17
**Figure S1**	Evaluation of time-dependent (5–45 min) release of glucose (**Glc**), maltose (**Mal**), and maltotriose (**Mal3**) after nanoGIT amylolysis (α-amylase, 5 µg/band and soluble starch solution, 20 µg/band) on HPTLC silica gel 60 plates. Plates were developed with acetonitrile/water/2-propanol (3:1:1 *V*/*V*/*V*) up to 70 mm and derivatized with p-aminobenzoic acid reagent (*p*-ABA, **a**), detected at FLD 366 nm and densitometrically evaluated at 366/>400 nm (fluorescence measurement, slit 4.0 mm × 0.2 mm, mercury lamp) and subsequently with 2-naphthol reagent (**b**), detected at white-light illumination in remission-transmission and densitometrically evaluated at 500 nm (absorbance measurement, slit 4.0 mm × 0.2 mm, deuterium/tungsten lamp).	S20
**Figure S2**	Verification of successful α-amylase inhibition by the ATI-containing refined wheat extract (inhibition assay) on maltotriose (**Mal3**) release via nanoGIT amylolysis (α-amylase, 2.5 µg/band and soluble starch, 20 µg/band) on HPTLC silica gel 60 plates. The addition of flour extract caused an increase in maltose (**Mal**) but no change in glucose (**Glc**). Plates were developed with acetonitrile/water/2-propanol (3:1:1 *V*/*V*/*V*) up to 70 mm and derivatised with *p*-aminobenzoic acid reagent (**a**), detected at FLD 366 nm, and subsequently with 2-naphthol reagent (**b**), detected at white-light illumination in remission-transmission	S21

**Figure S3**	Removal of saccharides from whole wheat flour extract via dialysis over 2–12 h, separated on HPTLC silica gel 60 plates with acetonitrile/water/2-propanol 3:1:1 (*V*/*V*/*V*) up to 70 mm and derivatized with diphenylamine aniline reagent, detected at white-light illumination at remission-transmission.	S22
**Figure S4**	Spectrophotometric determination of the inhibitory potential of the positive control trypsin inhibitor (**A**, 0.03–8.3 µg/mL), refined wheat flour extract (**B**, 3–300 µL), whole wheat flour extract (**C**, 3–300 µL) and einkorn flour extract (**D**, 3–300 µL) on the spectrophotometric trypsin assay (0.0015 mg/mL trypsin and 0.15 mg/mL L-BAPA, E/S 1:100). Comparison of the handheld anvajo fluidlab and a tabletop photometer (Camspec M501).	S23
**Figure S5**	Evaluation of the ideal enzyme-inhibitor ratio (E/I, 1:0.04-3.1) for the positive control trypsin inhibitor (**TI**) in the in-vial trypsin inhibition assay (7 µL/band). As the negative control (**NC**) trypsin (0.02 mg/mL) and casein (2 mg/mL) were used. Additionally, trypsin (0.02 mg/mL), casein (2 mg/mL) and TI (0.01 mg/mL) were applied as blanks (7 µL/band) onto HPTLC plates silica gel 60, developed with 2-butanol/pyridine/ammonia (25%)/water 10:17:5:13 (*V*/*V*/*V*/*V*) up to 50 mm, derivatized with the ninhydrin reagent and detected at white-light illumination in remission-transmission.	S24
**Figure S6**	Evaluation of the inhibitory potential of different volumes of refined wheat flour extract (100–175 µL) and pre-incubation periods (10-30 min) on in-vial trypsin-casein (**NC**, 1:100) digestion (7 µL/band). Additionally, the digestibility of the refined wheat extract by trypsin was evaluated. All were separated on HPTLC silica gel 60 plates with 2-butanol/pyridine/ammonia (25%)/water 10:17:5:13 (*V*/*V*/*V*/*V*) up to 50 mm, derivatised with the ninhydrin reagent, and detected at white-light illumination in remission-transmission.	S25
**Figure S7**	Repetition of the in-vial trypsin inhibition assay (7 µL/band), followed by HPTLC analysis: HPTLC–Vis chromatograms showing inhibition (framed black) of proteolysis (marked **+**) by three flour extracts (100 and 150 µL each) as well as PC trypsin inhibitor (**TI**, 1:2–3) mixed in a vial with trypsin and casein (**NC**, 1:100), pre-incubated (10 min), and incubated (30 min) at 37 °C. Additionally, non-proteolyzed (marked **−**) flour extract, TI, and trypsin were applied and separated on an HPTLC silica gel 60 plate with 2-butanol/pyridine/ammonia (25%)/water 10:17:5:13 (*V*/*V*/*V*/*V*) up to 50 mm, derivatised with ninhydrin reagent, and detected under white-light illumination (remission-transmission).	S26
**Figure S8**	Evaluation of the optimal on-surface enzyme-substrate ratio (E/S, 1:50-100) for the negative control (**NC**) of the HPTLC–nanoGIT (proteolysis inhibition)–Vis on HPTLC silica gel 60 plates with different absolute amounts (in µg/band) of trypsin and casein, respectively: 0.8:40 (**a**), 0.2:20 (**b**), 0.4:40 (**c**), 0.8:80 (**d**). Additionally, a casein blank (40 and 80 µg/band), which was either dried by a plate heater (**Heat**, **a–d**) or by a hair dryer (**No heat**) and an **in-vial** NC (7 µL/band, 1:100, 0.02 µg/µL trypsin, 2 µg/µL casein) was applied. All plates were separated with 2-butanol/pyridine/ammonia (25%)/water 10:17:5:13 (*V*/*V*/*V*/*V*) up to 50 mm, derivatised with the ninhydrin reagent and detected at white-light illumination in remission-transmission.	S27

**Figure S9**	HPTLC–nanoGIT (proteolysis inhibition)–Vis chromatograms showing the repetition of inhibition (framed black) of the proteolysis (marked **+**) by three flour extracts (2, 4 and 7 µL/band), trypsin inhibitor (**TI**, 1 µL/band, 0.5 mg/mL) as PC (E/I 1:2.5) and maximal proteolysis (**NC**, E/S 1:50) with trypsin (4 µL/band, 0.2 mg/mL) and casein (2 µL/band, 20 mg/mL) after 30 min incubation at 37 °C. Additionally, non-proteolyzed (marked **−**) flour extracts, TI and trypsin in the mentioned amount was applied and separated on HPTLC silica gel 60 plates with 2-butanol/pyridine/ammonia (25%)/water 10:17:5:13 (*V*/*V*/*V*/*V*) up to 50 mm, derivatised with the ninhydrin reagent, and detected under white-light illumination in remission-transmission.	S28

Table S1 Spectrophotometric α-amylase inhibition assay of wheat flour extract and acarbose as positive control evaluated by the handheld anvajo fluidlab and a tabletop photometer (Camspec M501).

Sample	Absorption	Corrected absorption	Inhibition [%]	Absorption	Corrected absorption	Inhibition [%]
	Anvajo fluidlab			Camspec M501		
Amylolysis	0.136			0.121		
Acarbose	1.746	1.669	92	1.591	1.473	92
Acarbose blank	0.077			0.118		
Wheat assay	1.010	0.472	71	1.426	0.479	75
Wheat blank	0.538			0.947		

Table S2 Evaluation of the on-surface α-amylase inhibition by acarbose and its metabolism by α-amylase for each saccharide described as the percentage deviation (Δ%) from amylolysis and α-amylase, respectively.

Substance	Saccharide	Intensity (AU)	Δ%
Inhibition by acarbose			
Acarbose (1 µg)	Glucose	0.5312137	−1
	Maltose	0.3703932	−5
	Maltotriose	0.1202205	−6
Acarbose (2 µg)	Glucose	0.4695306	−12
	Maltose	0.3217580	−17
	Maltotriose	0.0973957	−24
Acarbose (5 µg)	Glucose	0.4070322	−24
	Maltose	0.2856623	−26
	Maltotriose	0.1239826	−4
Amylolysis	Glucose	0.5363443	
	Maltose	0.3886226	
	Maltotriose	0.1285642	
Acarbose metabolization			
Acarbose + amylase	Glucose	0.4016023	29
	Maltose	0.3592573	29
	Maltotriose	0.1228561	17
Amylase	Glucose	0.3103049	
	Maltose	0.2795097	
	Maltotriose	0.1046252	

Table S3 α-Amylase inhibition by acarbose in each HPTLC–nanoGIT (amylolysis inhibition)–FLD/Vis analysis and the difference between the derivatization reagents *p*-aminobenzoic acid and 2-naphthol and the determined precision (n = 3) as relative standard deviation (RSD) for each saccharide.

Analysis	Saccharide	*p*-Aminobenzoic acid	2-Naphthol	Difference Δ%
Inhibition by acarbose [%]				
Refined wheat	Glucose	19	35	16
	Maltose	65	58	7
	Maltotriose	85	71	13
Whole wheat	Glucose	12	29	17
	Maltose	50	46	3
	Maltotriose	82	65	17
Einkorn	Glucose	16	45	29
	Maltose	58	50	8
	Maltotriose	84	68	16
Interday precision (RSD) [%] (n = 3 plates)				
Acarbose	Glucose	21	23	2
	Maltose	13	11	2
	Maltotriose	2	5	3

Table S4 Determined signal height of each saccharide of the HPTLC–nanoGIT (amylolysis inhibition)–FLD/Vis analysis of refined wheat, corrected signal, and calculated relative inhibition of both derivatisation reagents.

***p*-Aminobenzoic acid**				
Sample	Saccharide	Signal height	Corrected signal	Inhibition [%]
Negative control				
Amylolysis	Glucose	0.52310		
	Maltose	0.25667		
	Maltotriose	0.13955		
Positive control				
Acarbose	Glucose	0.42478		19
	Maltose	0.09105		65
	Maltotriose	0.02110		85
Refined wheat extract				
2 µL	Glucose	0.52325	0.37877	28
	Maltose	0.28431	0.25147	2
	Maltotriose	0.10277	-	26
5 µL	Glucose	0.52354	0.22597	57
	Maltose	0.27168	0.19959	22
	Maltotriose	0.06793	-	51
7 µL	Glucose	0.53331	0.15408	71
	Maltose	0.26659	0.16878	34
	Maltotriose	0.06225	-	55
9 µL	Glucose	0.56442	0.15209	71
	Maltose	0.26778	0.15752	39
	Maltotriose	0.05554	-	60
11 µL	Glucose	0.56595	0.15214	71
	Maltose	0.26313	0.15018	41
	Maltotriose	0.04321	-	69
Refined wheat extract blanks				
2 µL	Glucose	0.14448		
	Maltose	0.03284		
	Maltotriose	-		

Sample	Saccharide	Signal height	Corrected signal	Inhibition [%]
Refined wheat extract blanks				
5 µL	Glucose	0.29757		
	Maltose	0.07209		
	Maltotriose	-		
7 µL	Glucose	0.37923		
	Maltose	0.09781		
	Maltotriose	-		
9 µL	Glucose	0.41233		
	Maltose	0.11026		
	Maltotriose	-		
11 µL	Glucose	0.41380		
	Maltose	0.11296		
	Maltotriose	-		
**2-Naphthol**				
Negative control				
Amylolysis	Glucose	0.14690		
	Maltose	0.38200		
	Maltotriose	0.36505		
Positive control				
Acarbose	Glucose	0.09571		35
	Maltose	0.16228		58
	Maltotriose	0.10439		71
Refined wheat extract				
2 µL	Glucose	0.12725	0.09062	38
	Maltose	0.42284	0.31913	16
	Maltotriose	0.30935	0.30935	15
5 µL	Glucose	0.14130	0.06248	57
	Maltose	0.40876	0.22485	41
	Maltotriose	0.25208	0.23455	36
7 µL	Glucose	0.15481	0.04752	68
	Maltose	0.40948	0.19625	49
	Maltotriose	0.22709	0.20895	43

Sample	Saccharide	Signal height	Corrected signal	Inhibition [%]
Refined wheat extract				
9 μL	Glucose	0.18953	0.06872	53
	Maltose	0.40767	0.17781	53
	Maltotriose	0.20885	0.18653	49
11 μL	Glucose	0.19999	0.07291	50
	Maltose	0.39942	0.16510	57
	Maltotriose	0.18621	0.16647	54
Refined wheat extract blanks				
2 μL	Glucose	0.03663		
	Maltose	0.10371		
	Maltotriose	-		
5 μL	Glucose	0.07882		
	Maltose	0.18391		
	Maltotriose	0.01752		
7 μL	Glucose	0.10729		
	Maltose	0.21323		
	Maltotriose	0.01815		
9 μL	Glucose	0.12081		
	Maltose	0.22986		
	Maltotriose	0.02232		
11 μL	Glucose	0.12708		
	Maltose	0.23432		
	Maltotriose	0.01974		

Table S5 Determined signal height of each saccharide of the HPTLC–nanoGIT (amylolysis inhibition)–FLD/Vis analysis of whole wheat, corrected signal, and calculated relative inhibition of both derivatisation reagents.

***p*-Aminobenzoic acid**				
Sample	Saccharide	Signal height	Corrected signal	Inhibition [%]
Negative control				
Amylolysis	Glucose	0.55529		
	Maltose	0.27225		
	Maltotriose	0.13754		
Positive control				
Acarbose	Glucose	0.48725		12
	Maltose	0.13686		50
	Maltotriose	0.02518		82
Whole wheat extract				
2 µL	Glucose	0.54721	0.23017	59
	Maltose	0.28660	0.23997	12
	Maltotriose	0.10427	-	24
5 µL	Glucose	0.55389	0.06232	89
	Maltose	0.28537	0.18097	34
	Maltotriose	0.08845	-	36
7 µL	Glucose	0.55766	0.01991	96
	Maltose	0.28671	0.14942	45
	Maltotriose	0.09000	-	35
9 µL	Glucose	0.55900	-0.00170	100
	Maltose	0.27987	0.11943	56
	Maltotriose	0.06958	-	49
11 µL	Glucose	0.53691	-0.01984	104
	Maltose	0.27635	0.08859	67
	Maltotriose	0.05492	0.05492	60
Whole wheat extract blanks				
2 µL	Glucose	0.31704		
	Maltose	0.04663		
	Maltotriose	-		

Sample	Saccharide	Signal height	Corrected signal	Inhibition [%]
Whole wheat extract blanks				
5 µL	Glucose	0.49157		
	Maltose	0.10440		
	Maltotriose	-		
7 µL	Glucose	0.53775		
	Maltose	0.13729		
	Maltotriose	-		
9 µL	Glucose	0.56070		
	Maltose	0.16044		
	Maltotriose	-		
11 µL	Glucose	0.55675		
	Maltose	0.18777		
	Maltotriose	-		
**2-Naphthol**				
Negative control				
Amylolysis	Glucose	0.16112		
	Maltose	0.39829		
	Maltotriose	0.37627		
Positive control				
Acarbose	Glucose	0.11459		29
	Maltose	0.21362		46
	Maltotriose	0.13236		65
Whole wheat extract				
2 µL	Glucose	0.15302	0.07101	56
	Maltose	0.43147	0.32440	19
	Maltotriose	0.30979	-	18
5 µL	Glucose	0.18604	0.03922	76
	Maltose	0.42666	0.24303	39
	Maltotriose	0.27268	0.24780	34
7 µL	Glucose	0.16725	0.00031	33
	Maltose	0.42592	0.22122	44
	Maltotriose	0.28054	0.25127	100

Sample	Saccharide	Signal height	Corrected signal	Inhibition [%]
Whole wheat extract				
9 µL	Glucose	0.27837	0.08974	44
	Maltose	0.41188	0.20091	50
	Maltotriose	0.23546	0.19990	47
11 µL	Glucose	0.28830	0.09786	39
	Maltose	0.39565	0.18286	54
	Maltotriose	0.19986	0.15969	58
Whole wheat extract blanks				
2 µL	Glucose	0.08201		
	Maltose	0.10707		
	Maltotriose	-		
5 µL	Glucose	0.14682		
	Maltose	0.18364		
	Maltotriose	0.02487		
7 µL	Glucose	0.16695		
	Maltose	0.20469		
	Maltotriose	0.02927		
9 µL	Glucose	0.18863		
	Maltose	0.21098		
	Maltotriose	0.03556		
11 µL	Glucose	0.19044		
	Maltose	0.21279		
	Maltotriose	0.04017		

Table S6 Determined signal height of each saccharide of the HPTLC–nanoGIT (amylolysis inhibition)–FLD/Vis analysis of einkorn, corrected signal, and calculated relative inhibition of both derivatisation reagents.

***p*-Aminobenzoic acid**				
Sample	Saccharide	Signal height	Corrected signal	Inhibition [%]
Negative control				
Amylolysis	Glucose	0.50952		
	Maltose	0.25346		
	Maltotriose	0.14310		
Positive control				
Acarbose	Glucose	0.42674		16
	Maltose	0.10645		58
	Maltotriose	0.02312		84
Einkorn extract				
2 µL	Glucose	0.50709	0.37536	26
	Maltose	0.27218	-	−7
	Maltotriose	0.12567	-	26
5 µL	Glucose	0.50502	0.19698	61
	Maltose	0.27515	0.23137	9
	Maltotriose	0.10163	-	29
7 µL	Glucose	0.49650	0.07770	85
	Maltose	0.27205	0.19956	21
	Maltotriose	0.11246	-	21
9 µL	Glucose	0.54581	0.15739	69
	Maltose	0.26060	0.19528	23
	Maltotriose	0.06772	-	53
11 µL	Glucose	0.54473	0.14338	72
	Maltose	0.24873	0.18413	27
	Maltotriose	0.04981	-	65
Einkorn extract blanks				
2 µL	Glucose	0.13174		
	Maltose	-		
	Maltotriose	-		

Sample	Saccharide	Signal height	Corrected signal	Inhibition [%]
Einkorn extract blanks				
5 µL	Glucose	0.30805		
	Maltose	0.04379		
	Maltotriose	-		
7 µL	Glucose	0.41880		
	Maltose	0.07249		
	Maltotriose	-		
9 µL	Glucose	0.38842		
	Maltose	0.06531		
	Maltotriose	-		
11 µL	Glucose	0.40136		
	Maltose	0.06460		
	Maltotriose	-		
**2-Naphthol**				
Negative control				
Amylolysis	Glucose	0.16857		
	Maltose	0.41597		
	Maltotriose	0.40387		
Positive control				
Acarbose	Glucose	0.09202		45
	Maltose	0.20803		50
	Maltotriose	0.12999		68
Einkorn extract				
2 µL	Glucose	0.13016	0.09609	43
	Maltose	0.44015	0.39991	4
	Maltotriose	0.37258	-	8
5 µL	Glucose	0.13744	0.06252	63
	Maltose	0.44478	0.35428	15
	Maltotriose	0.32820	0.31253	23
7 µL	Glucose	0.12854	0.01738	90
	Maltose	0.44614	0.31789	24
	Maltotriose	0.35679	0.33245	18

Sample	Saccharide	Signal height	Corrected signal	Inhibition [%]
Einkorn extract				
9 μL	Glucose	0.17582	0.08109	52
	Maltose	0.39746	0.28085	32
	Maltotriose	0.25117	0.22598	44
11 μL	Glucose	0.17398	0.07694	54
	Maltose	0.37629	0.25330	39
	Maltotriose	0.22583	0.20028	50
Einkorn extract blanks				
2 μL	Glucose	0.03407		
	Maltose	0.04023		
	Maltotriose	-		
5 μL	Glucose	0.07492		
	Maltose	0.09050		
	Maltotriose	0.01567		
7 μL	Glucose	0.11116		
	Maltose	0.12825		
	Maltotriose	0.02434		
9 μL	Glucose	0.09473		
	Maltose	0.11661		
	Maltotriose	0.02519		
11 μL	Glucose	0.09704		
	Maltose	0.12300		
	Maltotriose	0.02555		

Table S7 Spectrophotometric trypsin inhibition assay with trypsin inhibitor and flour extracts of refined wheat, whole wheat, and einkorn.

Sample	Absorption	Inhibition [%]	Inhibition/µL	Absorption	Inhibition [%]	Inhibition/µL
	Anvajo fluidlab			Camspec M501		
Trypsin inhibitor #1						
Trypsin blank	0.012			0.015		
Proteolysis	0.127			0.125		
65 µL blank	0.018	84.3	1.3	0.008	83.6	1.3
65 µL	0.036			0.026		
30 µL blank	0.014	47.0	1.6	0.008	36.4	1.2
30 µL	0.075			0.078		
3 µL blank	0.011	9.6	3.2	0.008	5.5	1.8
3 µL	0.115			0.112		
1 µL blank	0.011	10.4	10.4	0.013	8.2	8.2
1 µL	0.114			0.114		
Trypsin inhibitor #2						
Trypsin blank	-0.0400			0.005		
Proteolysis	0.1240			0.292		
300 µL blank	-0.0364			0.014	95.1	0.3
300 µL	-0.0257			0.028		
100 µL blank	-0.0487			0.011	95.8	1.0
100 µL	-0.0170			0.023		
Refined wheat extract						
Trypsin blank	0.020			0.011		
Proteolysis	0.137			0.125		
300 µL blank	0.028	98.3	0.3	0.011	88.6	0.3
300 µL	0.030			0.024		
150 µL blank	0.024	80.3	0.5	0.012	76.3	0.5
150 µL	0.047			0.039		
100 µL blank	0.023	59.8	0.6	0.012	62.3	0.6
100 µL	0.070			0.055		
80 µL blank	0.024	58.1	0.7	0.013	56.1	0.7
80 µL	0.073			0.063		
60 µL blank	0.022	42.7	0.7	0.012	32.5	0.5
60 µL	0.089			0.089		

Sample	Absorption	Inhibition [%]	Inhibition/µL	Absorption	Inhibition [%]	Inhibition/µL
	Anvajo fluidlab			Camspec M501		
50 µL blank	0.023	28.2	0.6	0.013	28.9	0.6
50 µL	0.107			0.094		
30 µL blank	0.021	0.0	0.0	0.016	0.0	0.0
30 µL	0.138			0.130		
15 µL blank	0.021	-15.4	-1.0	0.013	-19.3	-1.3
15 µL	0.156			0.149		
10 µL blank	0.022	-13.7	-1.4	0.012	-22.8	-2.3
10 µL	0.155			0.152		
5 µL blank	0.022	-21.4	-4.3	0.013	-23.7	-4.7
5 µL	0.164			0.154		
3 µL blank	0.021	-36.8	-12.3	0.013	-33.3	-11.1
3 µL	0.181			0.165		
Whole wheat extract						
Trypsin blank	0.017			0.010		
Proteolysis	0.231			0.199		
300 µL blank	0.044	94.4	0.3	0.039	92.6	0.3
300 µL	0.056			0.053		
150 µL blank	0.023	83.6	0.6	0.012	79.9	0.5
150 µL	0.058			0.050		
100 µL blank	0.026	72.0	0.7	0.015	66.7	0.7
100 µL	0.086			0.078		
80 µL blank	0.024	63.1	0.8	0.014	59.3	0.7
80 µL	0.103			0.091		
60 µL blank	0.024	39.3	0.7	0.013	32.8	0.5
60 µL	0.154			0.140		
50 µL blank	0.022	27.1	0.5	0.014	21.7	0.4
50 µL	0.178			0.162		
30 µL blank	0.021	7.0	0.2	0.014	-0.5	0.0
30 µL	0.220			0.204		
15 µL blank	0.020	1.9	0.1	0.010	-6.3	-0.4
15 µL	0.230			0.211		
10 µL blank	0.020	-1.9	-0.2	0.012	-9.5	-1.0

Sample	Absorption	Inhibition [%]	Inhibition/µL	Absorption	Inhibition [%]	Inhibition/µL
	Anvajo fluidlab			Camspec M501		
10 µL	0.238			0.219		
5 µL blank	0.021	-1.9	-0.4	0.010	-11.6	-2.3
5 µL	0.239			0.221		
3 µL blank	0.022	-5.1	-1.7	0.012	-12.7	-4.2
3 µL	0.247			0.225		
Einkorn extract						
Trypsin blank	0.019			0.009		
Proteolysis	0.217			0.194		
300 µL blank	0.062	81.3	0.3	0.053	80.5	0.3
300 µL	0.099			0.089		
150 µL blank	0.039	87.9	0.6	0.028	82.7	0.6
150 µL	0.063			0.060		
100 µL blank	0.043	93.9	0.9	0.031	94.6	0.9
100 µL	0.055			0.041		
80 µL blank	0.049	97.5	1.2	0.031	95.1	1.2
80 µL	0.054			0.04		
60 µL blank	0.027	68.7	1.1	0.016	68.6	1.1
60 µL	0.089			0.074		
50 µL blank	0.028	52.5	1.1	0.015	51.4	1.0
50 µL	0.122			0.105		
30 µL blank	0.023	14.6	0.5	0.015	13.0	0.4
30 µL	0.192			0.176		
15 µL blank	0.020	-5.1	-0.3	0.010	-2.2	-0.1
15 µL	0.228			0.199		
10 µL blank	0.020	-7.6	-0.8	0.009	-11.9	-1.2
10 µL	0.233			0.216		
5 µL blank	0.019	-15.2	-3.0	0.007	-16.2	-3.2
5 µL	0.247			0.222		
3 µL blank	0.017	-15.2	-5.1	0.007	-16.8	-5.6
3 µL	0.245			0.223		

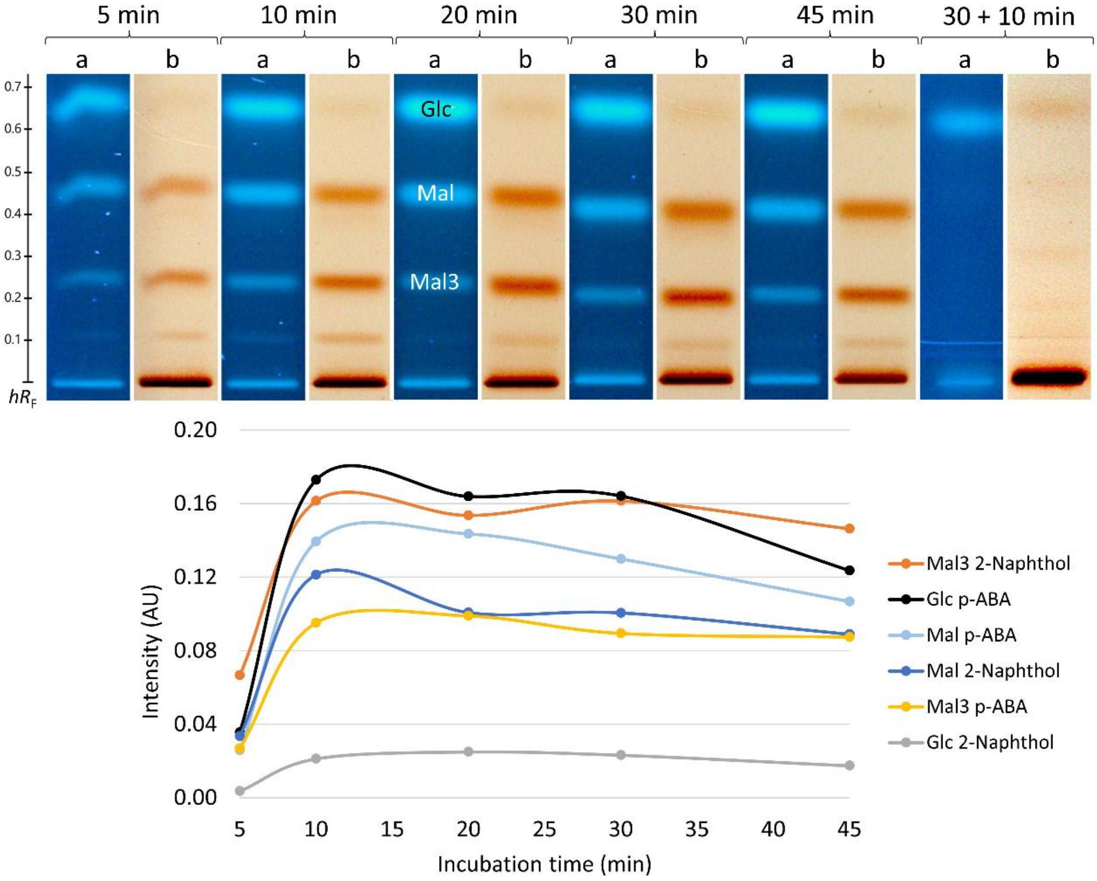

Figure S1 Evaluation of time-dependent (5–45 min) release of glucose (**Glc**), maltose (**Mal**), and maltotriose (**Mal3**) after nanoGIT amylolysis (α-amylase, 5 µg/band and soluble starch solution, 20 µg/band) on HPTLC silica gel 60 plates. Plates were developed with acetonitrile/water/2-propanol (3:1:1 *V*/*V*/*V*) up to 70 mm and derivatized with p-aminobenzoic acid reagent (*p*-ABA, **a**), detected at FLD 366 nm and densitometrically evaluated at 366/>400 nm (fluorescence measurement, slit 4.0 mm × 0.2 mm, mercury lamp) and subsequently with 2-naphthol reagent (**b**), detected at white-light illumination in remission-transmission and densitometrically evaluated at 500 nm (absorbance measurement, slit 4.0 mm × 0.2 mm, deuterium/tungsten lamp).

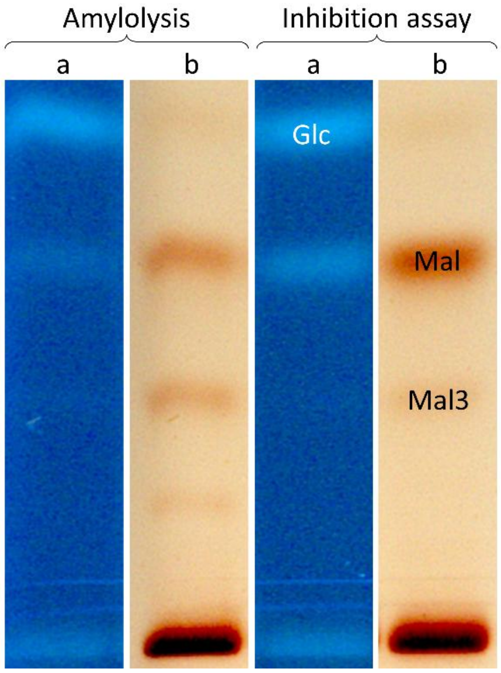

Figure S2 Verification of successful α-amylase inhibition by the ATI-containing refined wheat extract (inhibition assay) on maltotriose (**Mal3**) release via nanoGIT amylolysis (α-amylase, 2.5 µg/band and soluble starch, 20 µg/band) on HPTLC silica gel 60 plates. The addition of flour extract caused an increase in maltose (**Mal**) but no change in glucose (**Glc**). Plates were developed with acetonitrile/water/2-propanol (3:1:1 *V*/*V*/*V*) up to 70 mm and derivatised with *p*-aminobenzoic acid reagent (**a**), detected at FLD 366 nm, and subsequently with 2-naphthol reagent (**b**), detected at white-light illumination in remission-transmission.

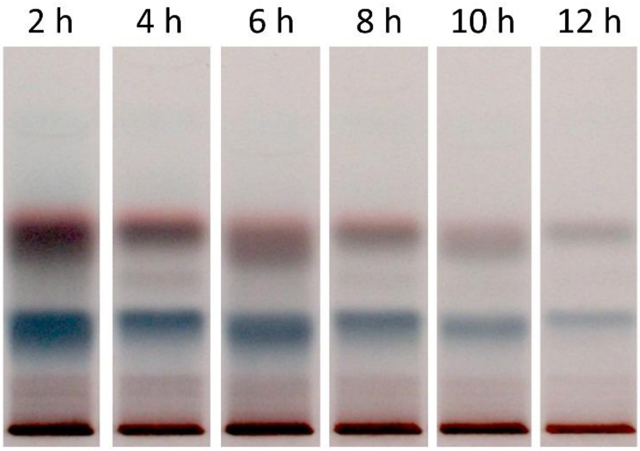

Figure S3 Removal of saccharides from whole wheat flour extract via dialysis over 2–12 h, separated on HPTLC silica gel 60 plates with acetonitrile/water/2-propanol 3:1:1 (*V*/*V*/*V*) up to 70 mm and derivatized with diphenylamine aniline reagent, detected at white-light illumination at remission-transmission.

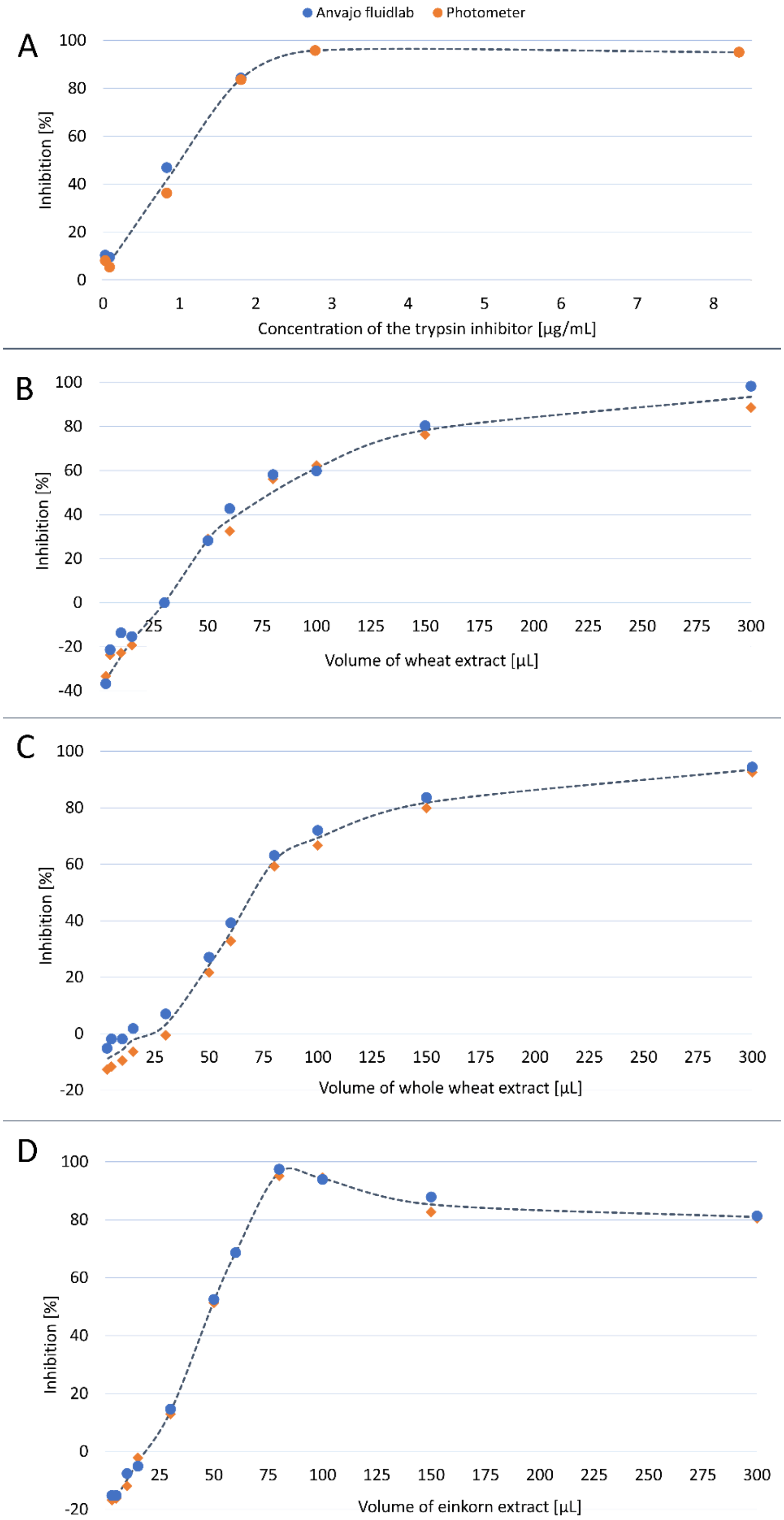

Figure S4 Spectrophotometric determination of the inhibitory potential of the positive control trypsin inhibitor (**A**, 0.03–8.3 µg/mL), refined wheat flour extract (**B**, 3–300 µL), whole wheat flour extract (**C**, 3–300 µL) and einkorn flour extract (**D**, 3–300 µL) on the spectrophotometric trypsin assay (0.0015 mg/mL trypsin and 0.15 mg/mL L-BAPA, E/S 1:100). Comparison of the handheld anvajo fluidlab and a tabletop photometer (Camspec M501).

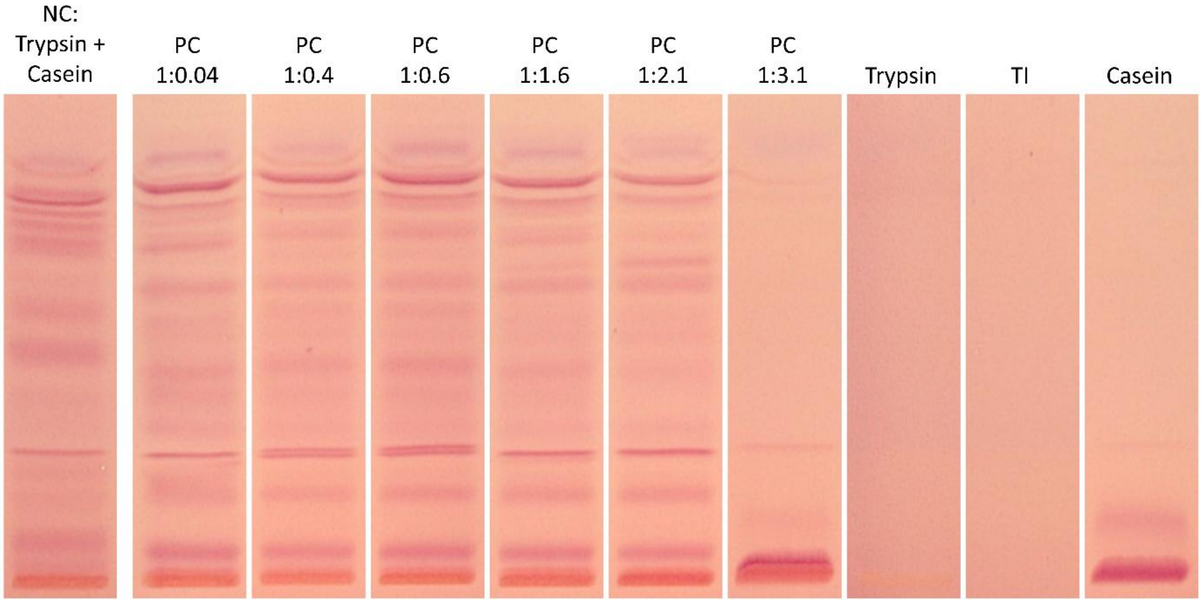

Figure S5 Evaluation of the ideal enzyme-inhibitor ratio (E/I, 1:0.04-3.1) for the positive control trypsin inhibitor (**TI**) in the in-vial trypsin inhibition assay (7 µL/band). As the negative control (**NC**) trypsin (0.02 mg/mL) and casein (2 mg/mL) were used. Additionally, trypsin (0.02 mg/mL), casein (2 mg/mL) and TI (0.01 mg/mL) were applied as blanks (7 µL/band) onto HPTLC plates silica gel 60, developed with 2-butanol/pyridine/ammonia (25%)/water 10:17:5:13 (*V*/*V*/*V*/*V*) up to 50 mm, derivatized with the ninhydrin reagent and detected at white-light illumination in remission-transmission.

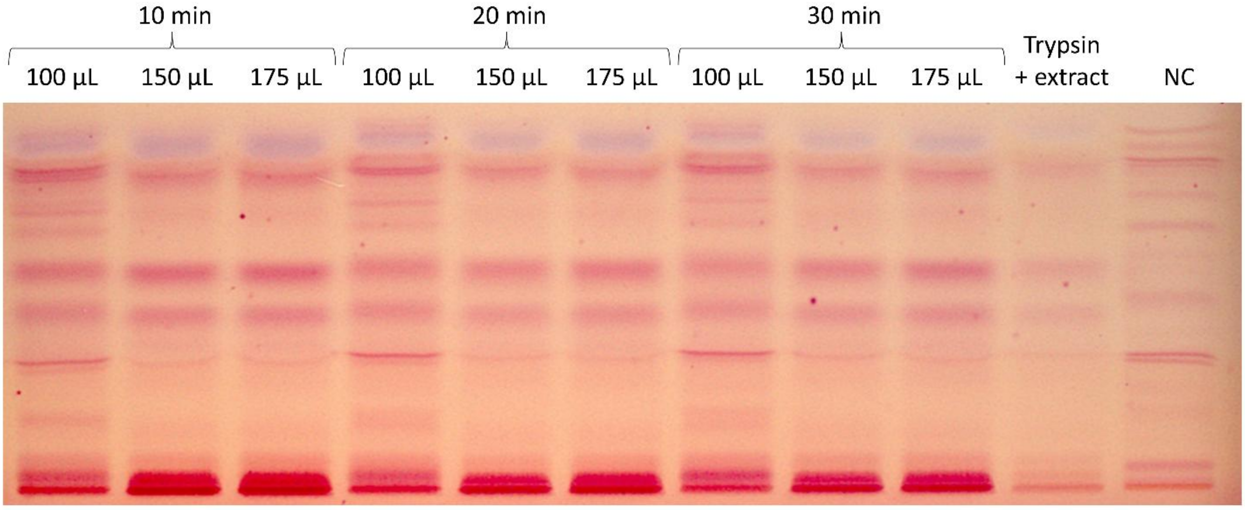

Figure S6 Evaluation of the inhibitory potential of different volumes of refined wheat flour extract (100–175 µL) and pre-incubation periods (10-30 min) on in-vial trypsin-casein (**NC**, 1:100) digestion (7 µL/band). Additionally, the digestibility of the refined wheat extract by trypsin was evaluated. All were separated on HPTLC silica gel 60 plates with 2-butanol/pyridine/ammonia (25%)/water 10:17:5:13 (*V*/*V*/*V*/*V*) up to 50 mm, derivatised with the ninhydrin reagent, and detected at white-light illumination in remission-transmission.

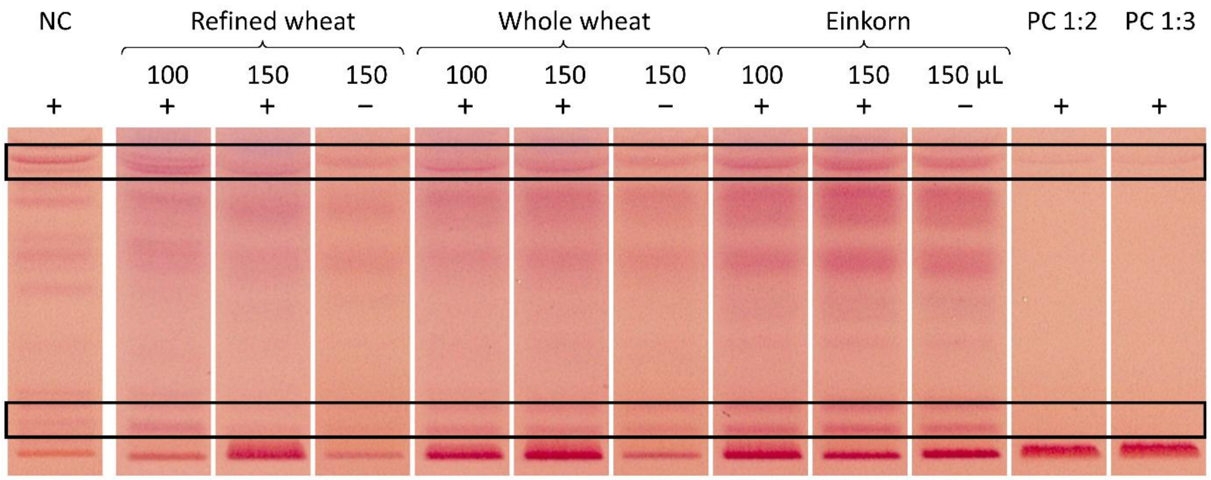

Figure S7 Repetition of the in-vial trypsin inhibition assay (7 µL/band), followed by HPTLC analysis: HPTLC–Vis chromatograms showing inhibition (framed black) of proteolysis (marked **+**) by three flour extracts (100 and 150 µL each) as well as PC trypsin inhibitor (**TI**, 1:2–3) mixed in a vial with trypsin and casein (**NC**, 1:100), pre-incubated (10 min), and incubated (30 min) at 37 °C. Additionally, non-proteolyzed (marked **−**) flour extract, TI, and trypsin were applied and separated on an HPTLC silica gel 60 plate with 2-butanol/pyridine/ammonia (25%)/water 10:17:5:13 (*V*/*V*/*V*/*V*) up to 50 mm, derivatised with ninhydrin reagent, and detected under white-light illumination (remission-transmission).

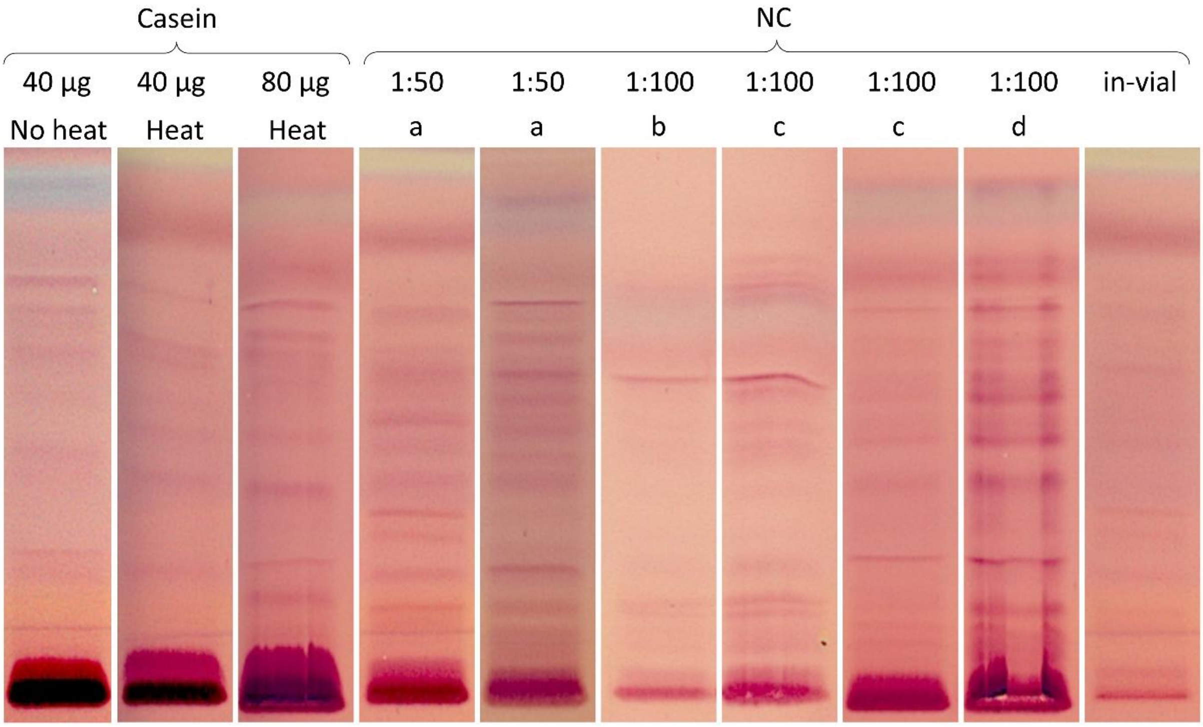

Figure S8 Evaluation of the optimal on-surface enzyme-substrate ratio (E/S, 1:50-100) for the negative control (**NC**) of the HPTLC–nanoGIT (proteolysis inhibition)–Vis on HPTLC silica gel 60 plates with different absolute amounts (in µg/band) of trypsin and casein, respectively: 0.8:40 (**a**), 0.2:20 (**b**), 0.4:40 (**c**), 0.8:80 (**d**). Additionally, a casein blank (40 and 80 µg/band), which was either dried by a plate heater (**Heat**, **a**–**d**) or by a hair dryer (**No heat**) and an **in-vial** NC (7 µL/band, 1:100, 0.02 µg/µL trypsin, 2 µg/µL casein) was applied. All plates were separated with 2-butanol/pyridine/ammonia (25%)/water 10:17:5:13 (*V*/*V*/*V*/*V*) up to 50 mm, derivatised with the ninhydrin reagent and detected at white-light illumination in remission-transmission.

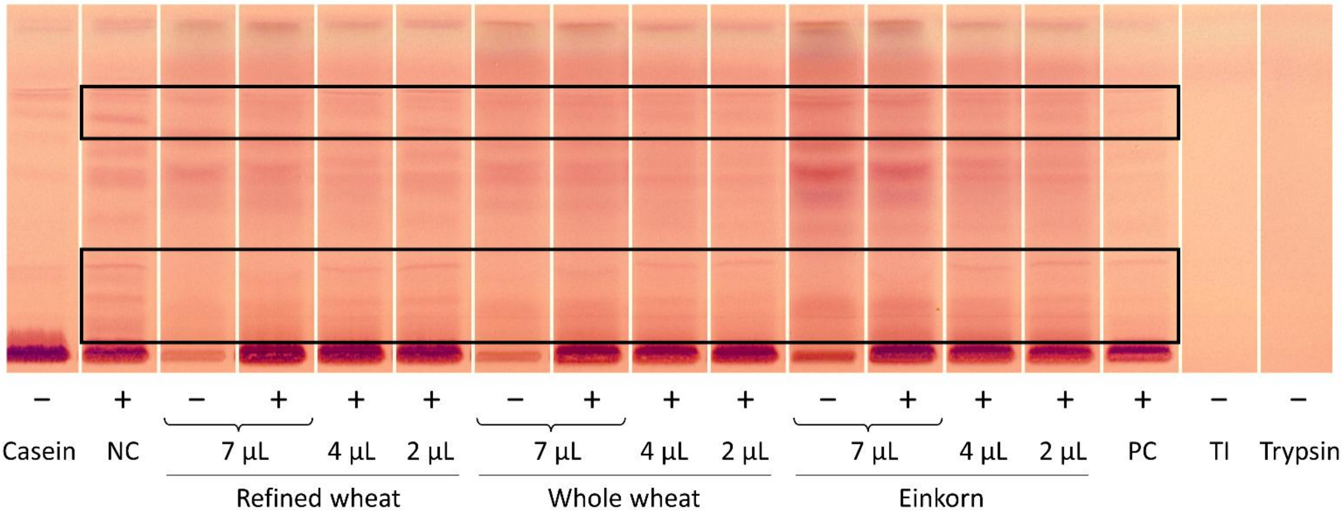

Figure S9 HPTLC–nanoGIT (proteolysis inhibition)–Vis chromatograms showing the repetition of inhibition (framed black) of the proteolysis (marked **+**) by three flour extracts (2, 4 and 7 µL/band), trypsin inhibitor (**TI**, 1 µL/band, 0.5 mg/mL) as PC (E/I 1:2.5) and maximal proteolysis (**NC**, E/S 1:50) with trypsin (4 µL/band, 0.2 mg/mL) and casein (2 µL/band, 20 mg/mL) after 30 min incubation at 37 °C. Additionally, non-proteolyzed (marked **−**) flour extracts, TI and trypsin in the mentioned amount was applied and separated on HPTLC silica gel 60 plates with 2-butanol/pyridine/ammonia (25%)/water 10:17:5:13 (*V*/*V*/*V*/*V*) up to 50 mm, derivatised with the ninhydrin reagent, and detected under white-light illumination in remission-transmission.
